# Real-time monitoring of ruminal microbiota reveals their roles in dairy goats during subacute ruminal acidosis

**DOI:** 10.1038/s41522-021-00215-6

**Published:** 2021-05-14

**Authors:** Xiaodong Chen, Xiaodong Su, Jilong Li, Yuntian Yang, Peiyue Wang, Fang Yan, Junhu Yao, Shengru Wu

**Affiliations:** 1 College of Animal Science and Technology, Northwest A&F University, Yangling, Shaanxi China; 2 Center for Translational Microbiome Research, Department of Molecular, Tumor and Cell Biology, Karolinska Institutet, Stockholm, Sweden

**Keywords:** Microbiome, Infectious-disease diagnostics

## Abstract

Ruminal microbiota changes frequently with high grain diets and the occurrence of subacute ruminal acidosis (SARA). A grain-induced goat model of SARA, with durations of a significant decrease in the rumen pH value to less than 5.6 and an increase in the rumen lipopolysaccharides concentration, is constructed for real-time monitoring of bacteria alteration. Using 16 S rRNA gene sequencing, significant bacterial differences between goats from the SARA and healthy groups are identified at every hour for six continuous hours after feeding. Moreover, 29 common differential genera between two groups over 6 h after feeding are all related to the altered pH and lipopolysaccharides. Transplanting the microbiota from donor goats with SARA could induce colonic inflammation in antibiotic-pretreated mice. Overall, significant differences in the bacterial community and rumen fermentation pattern between the healthy and SARA dairy goats are real-time monitored, and then tested using ruminal microbe transplantation to antibiotic-treated mice.

## Introduction

The gastrointestinal tract is a microbial ecosystem that is teeming with microorganisms, including bacteria, fungi, protozoa, archaea, and viruses. The gastrointestinal microbiota has been functionally connected to digestion and absorption, metabolism, immune homeostasis, and the neuroendocrine regulation of the host^1–5^. The presence of rumen and ruminal microbiota allows ruminants to use the starch and other nonfibrous carbohydrates from grain, and more importantly, to employ fibrous carbohydrates more efficiently than monogastric animals^6,7^. The excess intake of grain within a short period can result in subacute rumen acidosis (SARA) in both dairy cows and goats^8,9^. A sharp drop in the rumen pH, a decrease in the abundance of cellulolytic bacteria, the damage of the rumen epithelial barrier, and the ultimate inflammatory response are always accompanied by the occurrence of SARA. Most previous studies have shown that the ruminal bacterial community changes along with the occurrence of SARA^10,11^. SARA is characterized by the presence of a ruminal pH <5.6 for more than 3 h per day^10,12^, but limited research has been performed using real-time monitoring of the ruminal bacterial community and ruminal environment alterations during the occurrence of SARA. However, this real-time monitoring might provide more information and help researchers identify the potential key genera involved in regulating the occurrence of SARA.

Most of the existing studies on rumen microorganisms are based on analyses of the correlations between the microbial flora and host phenotype, and there is no corresponding model animal to reveal the symbiotic relationship and causality between ruminal microorganisms and the symptom in ruminants during SARA^13–16^. By using model animals such as *Drosophila*, mice, zebrafish, etc., numerous studies have focused on the function of gastrointestinal microbiota and have revealed the causality between microbiota and their hosts^17–19^. Currently, fecal microbiota transplant (FMT) methods in laboratory model animals, such as germ-free or antibiotic-treated mice, are commonly used to study the causality between gut microbiota and host phenotype changes^20,21^. By transplanting the functional microbiota of the feces from patients and healthy people into the gastrointestinal tract of germ-free or antibiotic-treated mice to rebuild the new intestinal microbiota, the roles of the microbiota in regulating the occurrence of different diseases have been widely explored in both humans and monogastric domestic animals such as pigs^22–24^. In ruminants, a study conducted by Ma et al. (2018) indicated that an FMT from cows with mastitis to germ-free mice resulted in mastitis symptoms in the mammary glands and systemic inflammation in mice^25^. Therefore, ruminal microbe transplantation (RMT) to antibiotic-treated mice may also serve as a potential method for testing the roles of ruminal microbiota in regulating the occurrence of SARA.

We hypothesized that rumen microbe transplantation from SARA dairy goats to antibiotic-treated mice could transfer key bacteria and then induce intestinal symptoms similar to those of SARA, such as intestinal inflammation. To test this hypothesis, the dairy goat model with SARA was used for the first time to monitor the ruminal bacterial community alteration during the occurrence of SARA in real-time (6 consecutive hours after feeding) using 16 S rRNA gene sequencing. The roles of the ruminal bacterial community in the consequentially altered rumen lipopolysaccharide and inflammation of dairy goats with grain-induced subacute ruminal acidosis were further tested using RMT methods.

## Results

### Rumen fermentation was changed in SARA dairy goats when compared with those of healthy dairy goats

**Fig. 1:**
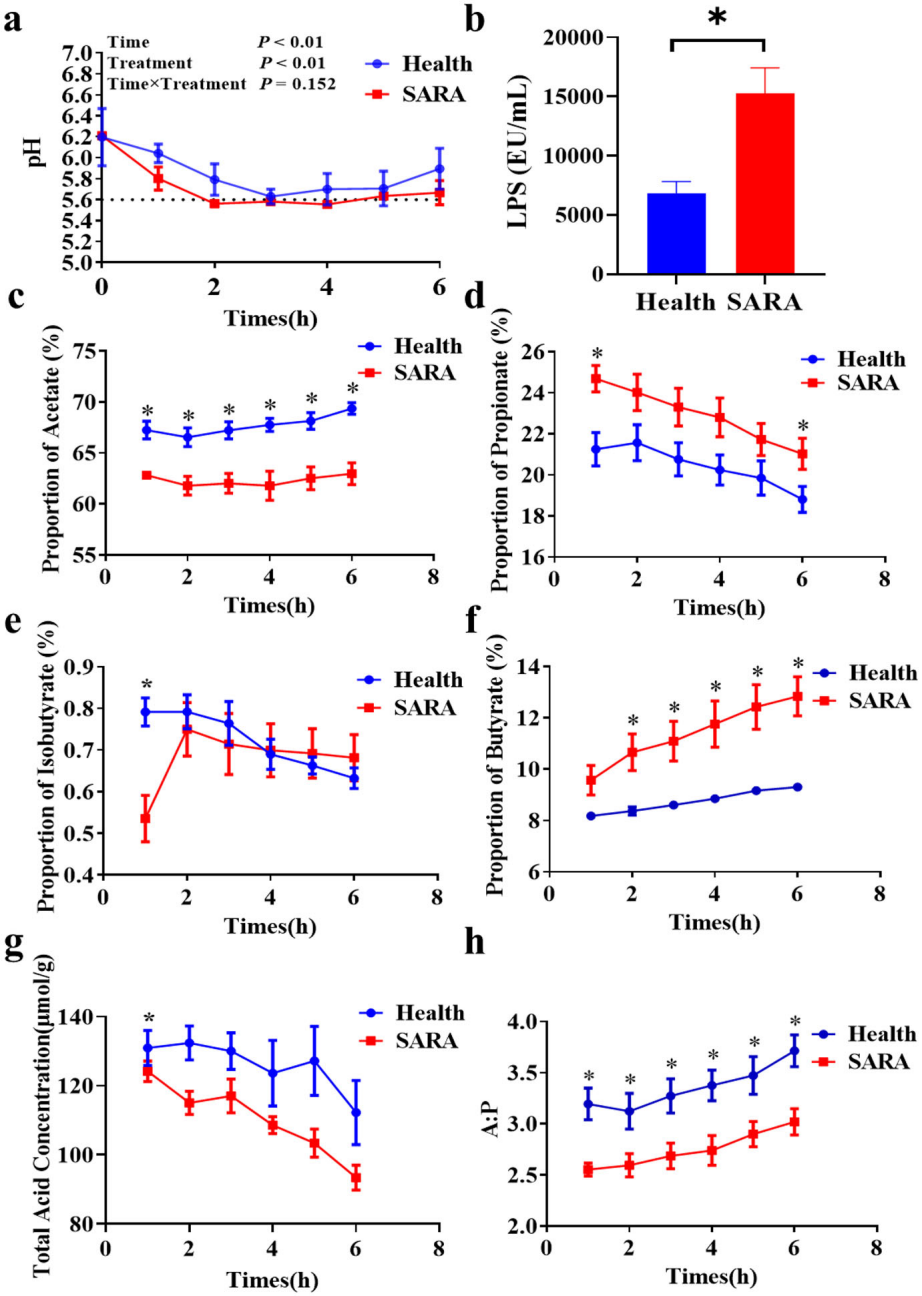
Rumen fermentation in two groups of dairy goat donors. **a** Changes in the rumen pH values of two groups of dairy goats 6 ho after feeding. **b** The difference in the LPS concentration in the rumen of two groups of dairy goats 2 h after feeding. **c**–**h** The proportions of (**c**) acetate, (**d**) propionate, (**e**) isobutyrate, (**f**) butyrate, (**g**) the ratio of acetate to propionate, and (**h**) the concentration of total volatile fatty acids of rumen fluid in two groups of dairy goats 6 h after feeding. The rumen pH values (**a**) were analyzed by the One-way Repeated Measures ANOVA procedure (the repeated measures analysis in the general linear model procedure). The other indices (**b**–**h**) were analyzed using the Students’ *t*-test. Health: the dairy goats in the healthy group, SARA: the dairy goats in the SARA group. *Indicates the difference is at a significant level with *p* < 0.05, **indicates the difference is at a significant level with *p* < 0.01. Error bars on the graphs represent standard error.

Within 6 h after feeding, compared with the dairy goats in the healthy group, the significantly increased duration in which the rumen pH value was less than 5.6 and the rumen lipopolysaccharides (LPS) concentration was significantly increased were identified in the SARA group (Fig. 1a, b). The proportion of acetate and the ratio of acetate to propionate in the rumen were significantly increased in goats from the healthy group after feeding. Moreover, significantly increased proportions of propionate and butyrate in the SARA group and significantly increased proportion of isobutyrate and concentrations of total volatile fatty acids in the healthy group were identified when compared with the other group at 1 h after feeding. However, no other significant change was identified between the SARA and healthy groups at the other time points (Fig. 1c–h). Furthermore, the proportions of valerate and isovalerate in the rumen were not significantly changed during all 6 h after feeding (Supplementary Fig. 1a, b).

### Rumen microbial composition of SARA dairy goats are distinct from those of healthy dairy goats

**Fig. 2:**
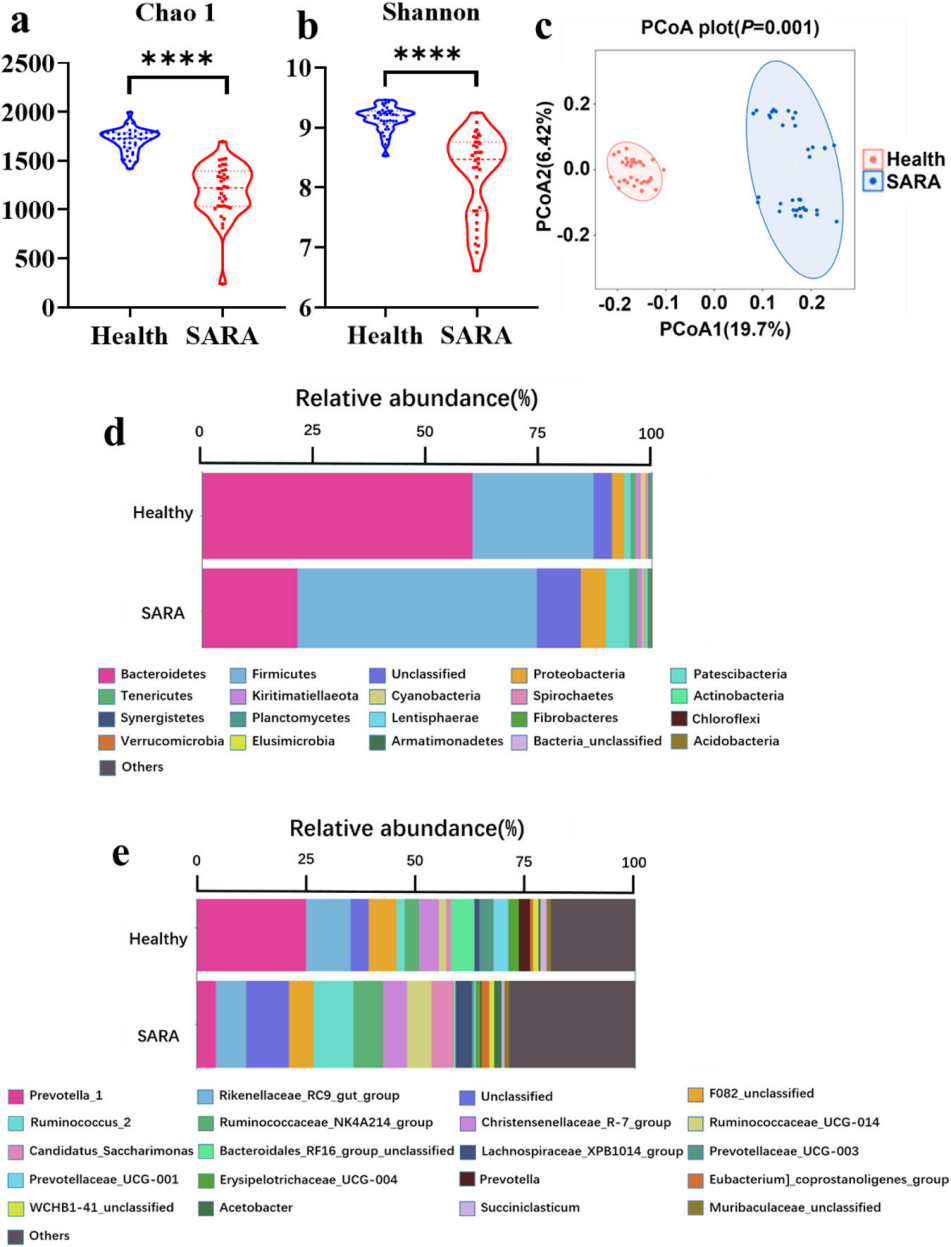
Rumen microbial composition of two groups of dairy goat donors. **a** The Chao 1 index and **b** the Shannon index of rumen microbiomes from two groups of dairy goat donors. A Mann–Whitney U test was carried out for comparing the two groups **a**, **b**. **c** Principal Coordinate Analysis (PCoA) of rumen microbiomes from two groups of dairy goat donors. The data was statistically analyzed based on ANOSIM analysis. **d** Average relative abundance of microbiota at the phylum level and **e** average relative abundance of microbiota at the genus level in SARA and healthy groups; Those bacteria whose relative abundance was less than 1% were classified as others. Health: rumen microorganisms from every hour for 6 consecutive hours after morning feeding of healthy dairy goats, SARA: rumen microorganisms from every hour for 6 consecutive hours after morning feeding of SARA dairy goats. *Indicates the difference is at a significant level with *p* < 0.05, **indicates the difference is at a significant level with *p* < 0.01. Error bars on the graphs represent standard error.

According to the alpha diversity analyses, the diversity and richness (Shannon and Chao 1 indices) of the rumen microbial communities in the healthy dairy goat group were all significantly increased. Further beta diversity analyses were performed, and the results showed that the SARA group was distinct from the healthy group (Fig. 2a–c). The differences in the bacteria between the two groups were analyzed. At the phylum level, a total of 27 bacterial phyla were identified in the two groups, and Bacteroidetes, Patescibacteria, Firmicutes, Proteobacteria, Kiritimatiellaeota, and Tenericutes were the common dominant phyla (relative abundance >1%) (Fig. 2d). Moreover, the significantly increased relative abundance of Firmicutes, Actinobacteria, Planctomycetes, Patescibacteria, Tenericutes, Armatimonadetes, and Chloroflexi and the significantly decreased relative abundance of Bacteroidetes, Cyanobacteria, Spirochaetes, Lentisphaerae, Fibrobacteres, and Kiritimatiellaeota were identified in the SARA group when compared with the healthy group (Supplementary Fig. 2a). At the genus level, a total of 527 bacterial genera were identified in the two groups, and *Prevotella 1, Ruminococcus 2, Candidatus Saccharimonas, Rikenellaceae RC9 gut group, F082 unclassified, Ruminococcaceae UCG-014, Eubacterium coprostanoligenes group, Lachnospiraceae XPB1014 group, Lachnospiraceae NK3A20 group, Ruminococcaceae NK4A214 group, Erysipelotrichaceae unclassified, Pseudobutyrivibrio, Mollicutes RF39 unclassified*, and *Christensenellaceae R-7 group* were the common dominant genera (relative abundance > 1%) (Fig. 2e). According to further analysis based on these dominant bacteria genera, the significantly altered genera were also identified. Compared with the healthy group, there were significantly increased relative abundances of *Ruminococcus 2, Ruminococcaceae NK4A214 group, Ruminococcaceae UCG-014, Candidatus Saccharimonas, Lachnospiraceae XPB1014 group, Eubacterium coprostanoligenes group, Lachnospiraceae NK3A20 group, Mollicutes RF39 unclassified, Pseudobutyrivibrio, Saccharofermentans*, and *Erysipelotrichaceae unclassified;* and there were significantly decreased relative abundances of *Prevotella 1, Rikenellaceae RC9 gut group, Bacteroidales RF16 group unclassified, Prevotellaceae UCG-003, Prevotellaceae UCG-001, Prevotella, Succiniclasticum*, and *Bacteroidales BS11 gut group unclassified* identified in the SARA group (Supplementary Fig. 2b).

### Dynamic changes in the differing common rumen bacteria during 6 h after feeding the donors from the two groups

**Fig. 3:**
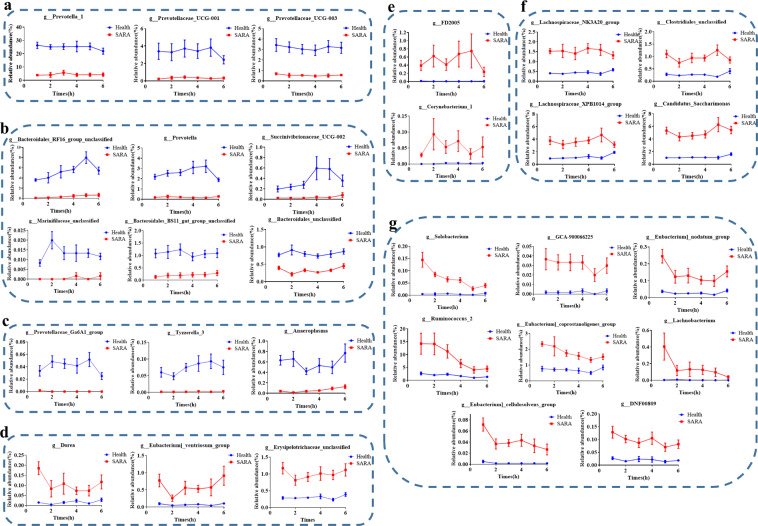
Dynamic changes in the common rumen differential bacteria (*P* < 0.05) during 6 h after feeding in donors from the SARA and healthy groups. **a** The relative abundance of the bacteria that increased significantly (FDR < 0.05) in the healthy group gradually decreased over time. **b** The relative abundance of the bacteria that increased significantly (FDR < 0.05) in the healthy group increased first and then decreased over time. **c** The relative abundance of the bacteria that increased significantly (FDR < 0.05) in the healthy group has unique changing laws over time. **d** The relative abundance of the bacteria that increased significantly (FDR < 0.05) in the SARA group changed with time and shows a “W” shape. **e** The relative abundance of the bacteria that increased significantly (FDR < 0.05) in the SARA group changed with time and displays an “M” shape. **f** The relative abundance of the bacteria that increased significantly (FDR < 0.05) in the SARA group decreased first, then increased and decreased over time. **g** The relative abundance of the bacteria that increased significantly (FDR < 0.05) in the SARA group gradually decreased over time. The Mann-Whitney U test was used with multiple comparisons adjusted by the Benjamini–Hochberg FDR to rank bacteria that were significantly different in their genus levels. All bacteria listed here were all significantly changed bacteria with *FDR* < 0.05 between SARA and Healthy groups in all 6 h after morning feeding. Health: the dairy goats from the healthy group, SARA: the dairy goats from the SARA group. Error bars on the graphs represent standard error.

We further analysed the rumen microbial changes at each time point over the 6 h after feeding, and we found that the diversity and richness (Shannon and Chao 1 indices) of the rumen microbial communities of dairy goats in the SARA group were all significantly decreased in comparison to the healthy group (Supplementary Figure 3a–l). There were significant differences in the bacterial community (Supplementary Fig. 4a–f) at each hour during the 6 h after feeding. In addition, we found 29 common differential genera during the 6 h after feeding in the goats from the two groups. Among the 12 genera that significantly increased in the healthy group, the relative abundance of *Prevotella 1, Prevotellaceae UCG-001*, and *Prevotellaceae UCG-003* were gradually decreased over the 6 h after feeding (Fig. 3a). However, the relative abundance of *Bacteroidales RF16 group unclassified*, *Prevotella*, and *Succinivibrionaceae UCG 002* (the relative abundance is highest 5 h after feeding) and the relative abundance of *Marinifilaceae unclassified, Bacteroidales BS11 gut group unclassified*, and *Bacteroidales unclassified* (Relative abundance is highest 2 h after feeding) were both first increased and then decreased over the 6 h after feeding (Fig. 3b). Moreover, the *Prevotellaceae Ga6A1 group* was first increased, then stable, and ultimately decreased, while the *Anaeroplasma* was first decreased, then stable, and ultimately increased. In addition, *Tyzzerella 3* first increased and then remained stable over the 6 h after feeding (Fig. 3c).

Among the 17 bacteria genera that increased significantly in the SARA group, the relative abundance of *Dorea, Eubacterium ventriosum group*, and *Erysipelotrichaceae unclassified* changed over the 6 h after feeding and showed a “W” shape (Fig. 3d), while the relative abundance of *Corynebacterium 1, FD2005* showed an “M” shape (Fig. 3e). Moreover, the relative abundance of *Lachnospiraceae XPB1014 group, Clostridiales unclassified, Lachnospiraceae NK3A20 group*, and *Candidatus Saccharimonas* were first decreased, then increased and ultimately decreased over the 6 h after feeding (Fig. 3f). In addition, the 8 other genera gradually decreased over the 6 h after feeding (Fig. 3g).

### Correlation analysis of different microorganisms at the genus level with rumen fermentation and the prediction of differential functions for rumen microbes between two groups of donors

**Fig. 4:**
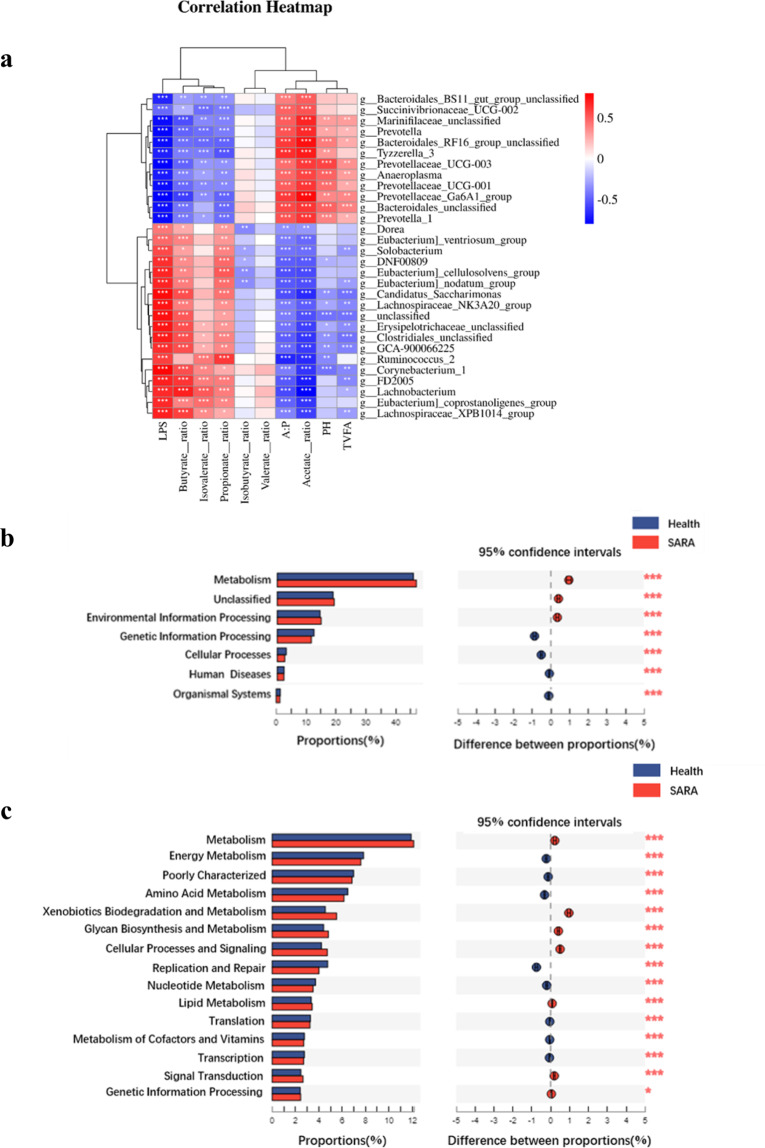
Correlation analysis and function prediction. **a** The Pearson correlation between the common genus-level differences in the bacteria during 6 h after feeding in donors from the two groups and their rumen fermentation parameters. **b** Prediction of the differential function of rumen microbes between two groups of dairy goat donors in multiple KEGG (level 1) categories based on PICRUSt 2. **c** Prediction of the differential function of rumen microbes between two groups of dairy goat donors in multiple KEGG (level 2) categories based on PICRUSt 2. Health: the dairy goats from the healthy group, SARA: the dairy goats of the SARA group. The Mann-Whitney U test was used with multiple comparisons adjusted by the Benjamini–Hochberg FDR to rank pathways that were significantly different in predicted metagenome pathways analysis. *Indicates the difference is at a significant level with *p* < 0.05, **indicates the difference is at a significant level with *p* < 0.01.

The 12 genera that significantly increased in the healthy group were significantly positively correlated with the ruminal pH, proportion of acetate, and concentration of total volatile fatty acids. The 17 genera that increased significantly in the SARA group were significantly positively correlated with the ruminal LPS concentration and the ruminal proportion of propionate, butyrate, and isovalerate (Fig. 4a).

The functional composition profiles of rumen microorganisms were predicted from 16 S rRNA sequencing data using PICRUSt2 (phylogenetic investigation of communities by reconstruction of unobserved states 2), and as shown in comparison with the healthy group, the SARA group had a significantly higher relative abundance for metabolism and environmental information processing genes but a lower relative abundance of genetic information processing, cellular processes, human diseases, and organismal systems in multiple KEGG (level 1) categories (Fig. 4b). Compared with the healthy group, the SARA group had a significantly lower relative abundance of metabolism of energy, amino acids, nucleotide cofactors and vitamins, and poorly characterized replication and repair, translation, and transcription genes, but it had a higher relative abundance of xenobiotics biodegradation and metabolism, glycan biosynthesis and metabolism, cellular processes and signalling, lipid metabolism, signal transduction, genetic information processing in multiple KEGG (level 2) categories (Fig. 4c).

### RMT could significantly influence the gut bacterial community colonization of pre-antibiotic-treated mice

After being treated with antibiotics, the microbial richness of the small intestine and colon in mice were significantly decreased (Supplementary Fig. 5a–d), and the bacterial community in the small intestine and colon were also significantly changed (Supplementary Fig. 5e, f). In addition, the numbers of amplicon sequence variants (ASVs) in the small intestine and colon of the mice were also significantly reduced after antibiotic treatment (Supplementary Fig. 5g, h), which indicated that the pre-antibiotic treatment could significantly deplete the original gut microbiota. Furthermore, the RMT with antibiotics pre-treatments could significantly alter both microbiota in the colon or small intestine of mice when compared with the mice without RMT and antibiotics (Supplementary Fig. 5i, j), but microbiota in the colon or small intestine of mice after the RMT without antibiotics pre-treatments could not significantly separate from the microbiota in the colon or small intestine of mice without RMT and antibiotics (Supplementary Fig. 5i, j). These results indicated that the antibiotics pre-treatment for RMT is essential, and also indicated that the RMT after antibiotics pre-treatment is succussed in the present study.

**Fig. 5:**
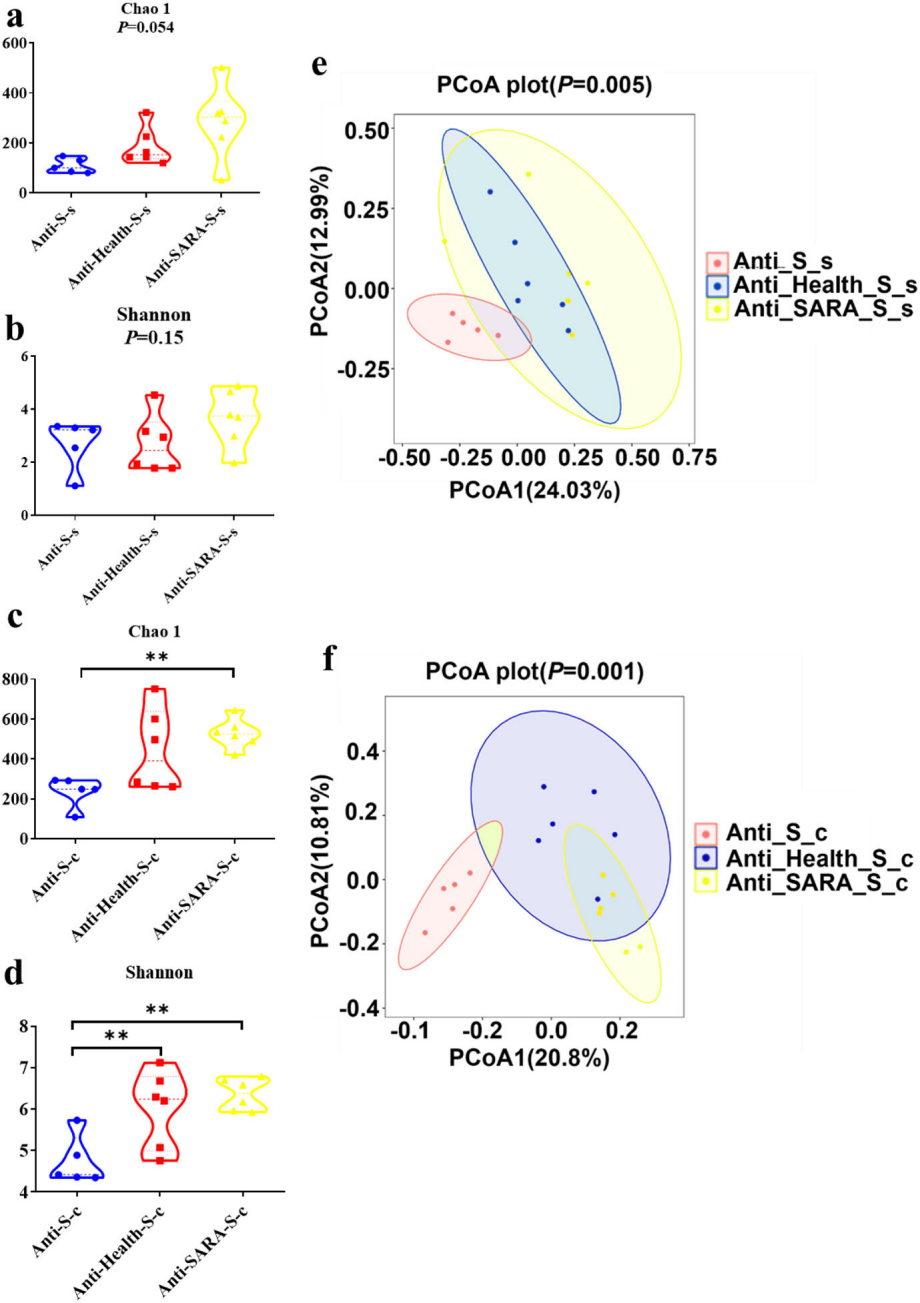
Effects of ruminal microbe transplantation (RMT) on the intestinal microbial composition of antibiotic-treated mice. **a** The Chao 1 index and **b** the Shannon index of the small intestine bacterial community after antibiotic-treated mice RMT. **c** The Chao 1 index and **d** the Shannon index of the colonic bacterial community after RMT in antibiotic-treated mice. The data of (**a**–**d**) were statistically analyzed using the Kruskal-Wallis test with Dunn’s post-hoc test. **e** Principal Coordinates Analysis of the small intestine bacterial community after RMT in antibiotic-treated mice. **f** Principal Coordinates Analysis of the colonic bacterial community after RMT in antibiotic-treated mice. The data of (**e**) and (**f**) were statistically analyzed based on ANOSIM analysis. Anti: mice taking antibiotics; Health: mice infused by intragastric gavage with rumen fluid from health dairy goats; SARA: mice infused by intragastric gavage with rumen fluid from SARA dairy goats; S: high starch diet for mice; s: small intestine of mice; and c: mice colon. *Indicates the difference is at a significant level with *p* < 0.05, **indicates the difference is at a significant level with *p* < 0.01.

According to the alpha diversity results for the colon microorganisms, compared with the Anti-S group that received antibiotics only and fed with a high starch diet, significantly increased Chao 1 and Shannon indices were identified in the Anti-SARA-S group that received both antibiotics and RMT from SARA goats and then fed with high starch diet. Notably, the significantly increased colon microbial Shannon index was also identified in the Anti-Health-S group that received both antibiotics and RMT from health goats and then fed with a high starch diet (Fig. 5c, d). However, there is no significant difference based on the alpha diversity of the small intestine microorganisms (Fig. 5a, b). The beta diversity analysis showed a significantly altered bacterial community in the small intestine and colon of mice after RMT as well (Fig. 5e, f). These results indicated that the RMT could significantly alter the gut bacterial community of pre-antibiotic-treated mice, especially the colon bacterial community.

When compared with the Anti-S group, significantly increased body weight gains were identified in the Anti-Health-S and Anti-SARA-S groups, and the significantly increased pancreas weight and pancreas index were identified in the Anti-Health-S group (Supplementary Table 1). These results may have been induced by the colonization of the microbiota after RMT, which also indicated that the RMT could influence the gut microbiota of the mice gut.

### Similar bacterial community and fermentation were identified between the colons of mice and the rumens of donor dairy goats

**Fig. 6:**
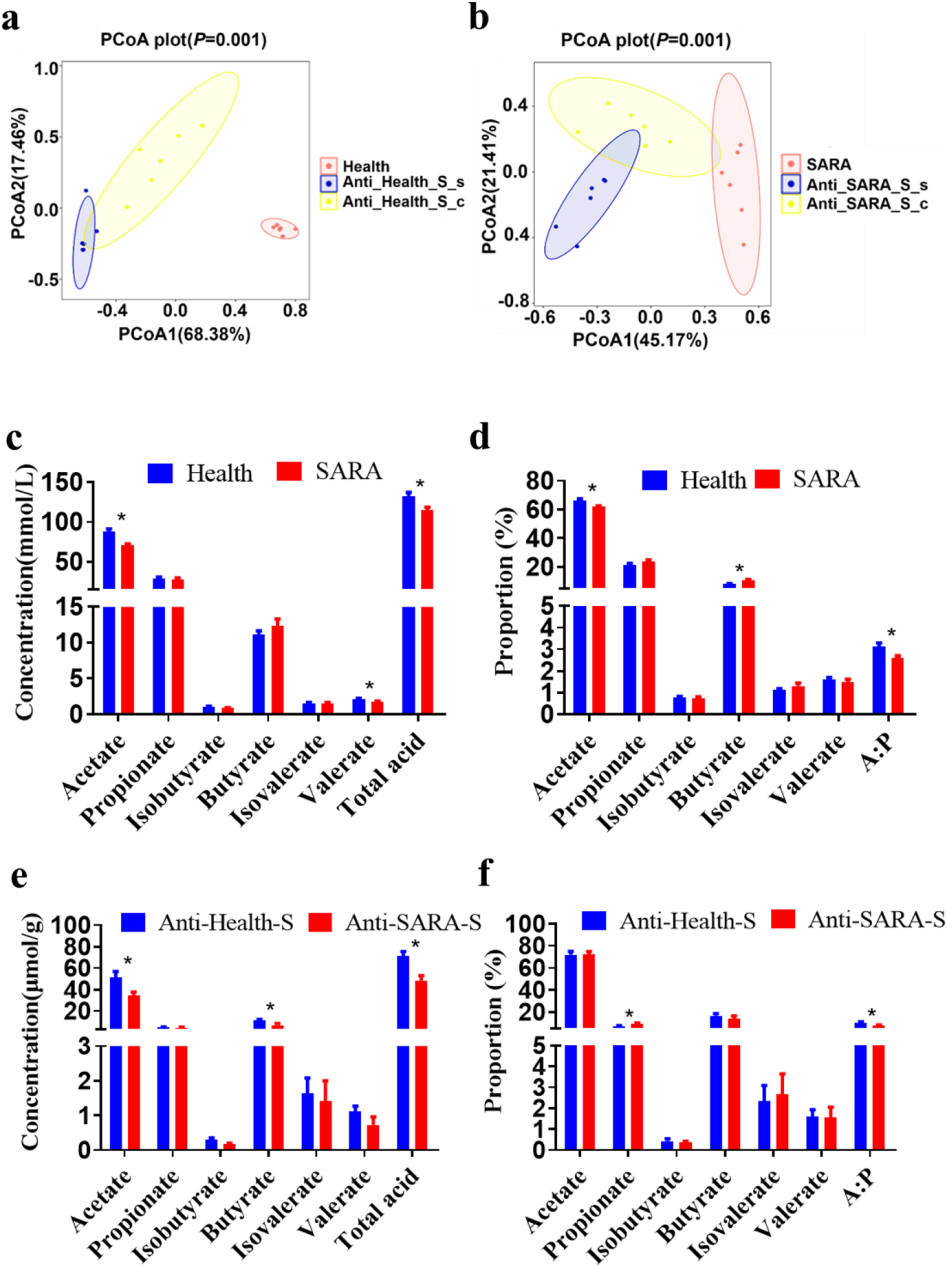
The intestinal microbial composition and intestinal fermentation parameters alterations of antibiotic-treated mice were identified after ruminal microbe transplantation (RMT). **a** Principal coordinate analysis of mouse (infused by intragastric gavage with rumen fluid from healthy dairy goats) intestinal bacterial community and donor (healthy dairy goats) rumen bacterial community based on the weighted UniFrac distance. **b** Principal Coordinate Analysis of mouse (infused by intragastric gavage with rumen fluid from SARA dairy goats) intestinal bacterial community and donor (SARA dairy goats) rumen bacterial community based on the weighted UniFrac distance. The data of (**a**) and (**b**) were statistically analyzed based on the ANOSIM analysis. **c** The concentration and **d** relative proportion of VFA in the rumen of healthy and SARA donors. **e** The concentration and **f** relative proportion of VFA in colons from mice given a high starch diet after RMT in antibiotic-treated mice. The data for (**c**–**f**) were analyzed using the Students’ t-test, and expressed as the means with the standard error. Anti: mice taking antibiotics; Health: mice infused by intragastric gavage with rumen fluid from healthy dairy goats; SARA: mice infused by intragastric gavage with rumen fluid from SARA dairy goats; S: high starch diet for mice; s: small intestine of mice; and c: mice colon. *Indicates the difference is at a significant level with *p* < 0.05, **indicates the difference is at a significant level with *p* < 0.01. Error bars on the graphs represent standard error.

According to the results of the beta diversity analysis, there were significant differences among the ruminal bacterial composition of goats, the colonic and intestinal microbial composition of the mice recipients (Fig. 6a, b). The weighted UniFrac ANOSIM distances of each two groups were further calculated. The distances between the ruminal bacterial composition of goats and colonic microbial composition of the mice recipient (including Health vs. Anti-Health-S-c groups, and SARA vs. Anti-SARA-S-c groups), were significantly lower than the distances between the ruminal bacterial composition of goats and small intestinal microbial composition of the mice recipient (Health vs. Anti-Health-S-s groups, and SARA vs. Anti-SARA-S-s groups) (*FDR* < 0.05, Supplementary Fig. 6a, b). This result indicated that the microbial composition of the donor’s rumen fluid was more similar to the colonic microbial composition of the mice recipients.

We further compared the VFA composition of each intestinal content of the mice with the VFA composition of the rumen fluid of the corresponding donor dairy goats. The results showed that when mice were fed a high-starch diet after RMT, only the composition of the VFA in the colon contents was significantly changed between mice from Anti-Health-S and Anti-SARA-S groups. Briefly, compared with the Anti-Health-S group, the concentrations of acetate, butyrate, total acid, and the ratio of acetate to propionate in the colon content in the Anti-SARA-S group were significantly reduced (Fig. 6e, f). Notably, the similar significant differences in ruminal acetate and total acid concentration and proportion were identified in goats from Health and SARA groups (Fig. 6c, d). However, the composition of VFAs in the contents of the small intestines and ceca of mice recipients were not significantly changed between Anti-Health-S and Anti-SARA S group after the RMT (Supplementary Fig. 6c–f).

### High-fibre diet can alleviate colonic inflammation in mice that mediated by rumen microorganisms of SARA dairy goats, and it can promote gene expression of colonic tight junction proteins

**Fig. 7:**
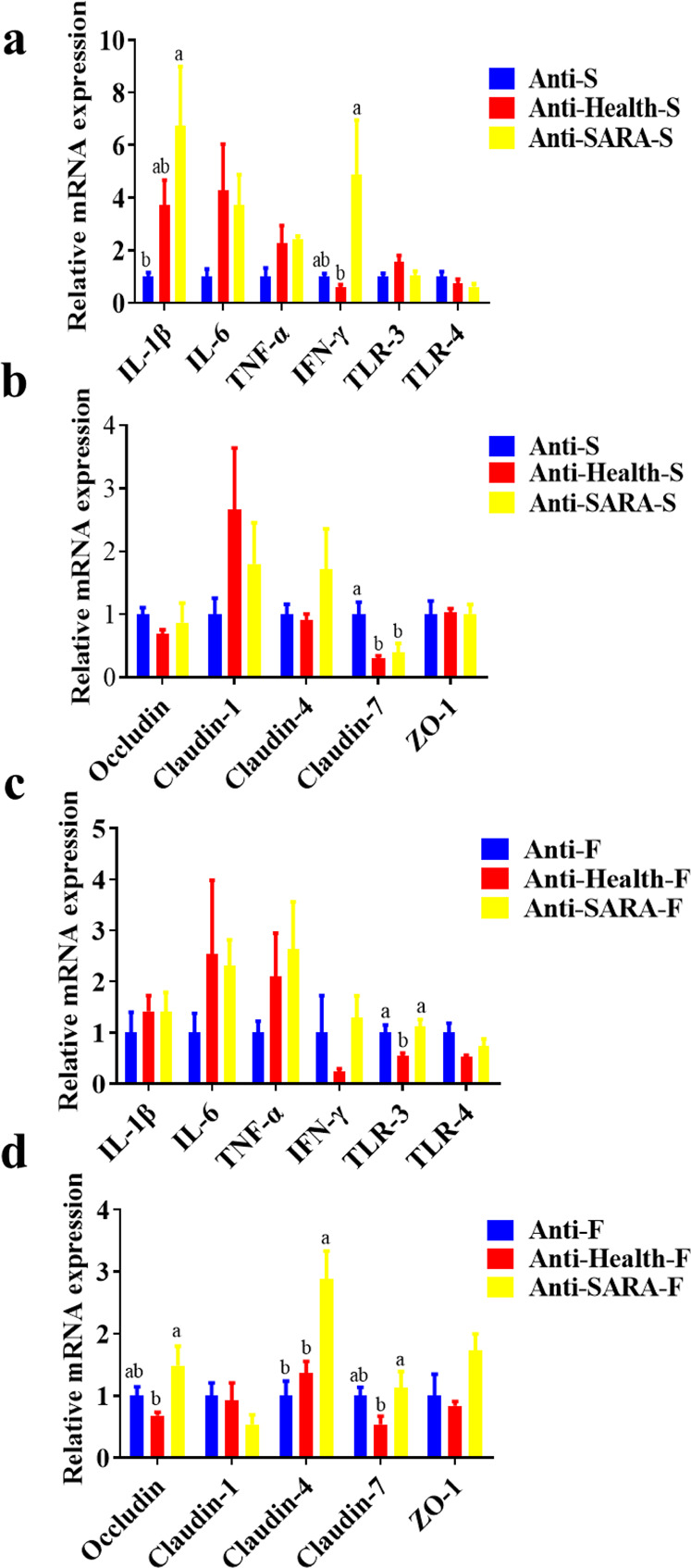
Effects of ruminal microbe transplantation (RMT) on colon inflammation and intestinal epithelial permeability of antibiotic-treated mice. **a** The relative mRNA expression of cytokines and **b** tight junction proteins in the colon epithelia in antibiotic-treated mice receiving a high starch diet after RMT. **c** The relative mRNA expression of cytokines and **d** tight junction proteins in the colon epithelia of antibiotic-treated mice fed with a high fibre diet after RMT. The data were analyzed using the ANOVA test. If a significant treatment effect was observed by ANOVA, the significant difference between treatments was identified by Duncan’s multiple comparisons test. Anti: mice taking antibiotics; Health: mice infused by intragastric gavage with rumen fluid from healthy dairy goats; SARA: mice infused by intragastric gavage with rumen fluid from SARA dairy goats; S: high starch diet for mice; F: high fibre diet for mice. ^a-b^ Mean values within an index with the same superscript letters indicated no significant difference (*P* < 0.05). Error bars on the graphs represent standard error.

Since the bacterial community and fermentation parameters of the mouse colon after RMT are more similar to those of the donor, we have also tested the inflammation indicators of the colon tissue and the permeability of the intestinal epithelium. Compared with the Anti-S group, the Anti-SARA-S group significantly increased the expression of *IL-1β* and *IFN-γ* mRNA in the mouse colon tissue, and both Anti-Health-S and Anti-SARA-S significantly decreased *Claudin-7* mRNA expression in the mouse colon (Fig. 7a, b).

In view of the mitigation effects of high-fibre diets on SARA in dairy goats, we conducted similar studies in recipient mice. The mice that consumed high-fibre diets after RMT showed that the inflammatory factors in colon tissue from the Anti-SARA-F (mice received both antibiotics and RMT from SARA goats and then fed with high fibre diet) were similar to that in Anti-Healthy-F (mice received both antibiotics and RMT from Healthy goats and then fed with high fibre diet) and Anti-F groups (that received antibiotics only and fed with high fibre diet). In addition, the Anti-SARA-F group significantly increased the mRNA expression of *Claudin-4* in mouse colon tissue when compared with the Anti-F group. Compared with Anti-Health-F, the Anti-SARA-F group significantly increased the mRNA expression of *Occludin*, *Claudin-4* and Claudin-7 in mouse colon tissue as well (Fig. 7c, d).

## Discussion

Rumen acidosis is the rapid fermentation process in ruminants after they take in a large amount of cereal feed, producing large amounts of organic acids when excessive levels of organic acids accumulate in the rumen, resulting in a rapid pH decrease in the rumen fluid. It is generally believed that subacute rumen acidosis occurs when the pH in the rumen is below 5.6 and the daily maintenance time is approximately 3 h, and acute acidosis occurs when the pH is below 5.0^10,12^. In our experiment, by changing the dietary composition, the SARA group maintained a pH of less than 5.6 for 3 h within 6 h after feeding, and it was believed that the SARA model contributed successfully. Carbohydrates are fermented by microorganisms in the rumen to produce a large amount of VFA. Therefore, when the dietary carbohydrate composition is changed, the VFA ratio will also change. There were significant differences in the rumen fermentation parameters between the healthy group and the SARA group in this experiment. Previous studies have shown that the fermentation of high-concentration diets increases the proportion of propionate in the rumen, and decreases the proportion of acetate and the ratio of acetate to propionate; the fermentation of low-concentration diets can increase the proportion of acetate^8,16^, which is consistent with the results of this study. Doepel et al.^26^ reported that the T-VFA concentration increased with the proportion of dietary wheat, and the change in the rumen pH was highly inversely related to the VFA concentration. However, this experiment showed that although the pH of the SARA group was reduced, the T-VFA concentration of the SARA group was significantly decreased, which may be related to the concentration of lactic acid in the rumen, the pH of which is much lower than that of the VFA, resulting in a lower rumen pH^27^, and the significant increase in the lactate-producing bacteria genera *Lachnospiraceae NK3A20 group* and *Lachnospiraceae XPB1014 group* in the SARA group also confirmed this result.

LPS is the primary component of the cell walls in gram-negative bacteria. A large number of studies have shown that when high-grain diets are fed to ruminants, gram-negative bacteria will proliferate in large numbers and then the bacteria will lyse, resulting in a sharp increase in the concentration of free LPS in the rumen^28,29^. The results of this study are consistent with previous studies in which the proportion of dietary concentrate was increased from 30% to 70%, and the concentration of free LPS in the rumen fluid increased from 6830 EU/mL to 15266 EU/mL. From the correlation analysis results of different bacteria and rumen fermentation indicators, it can be concluded that the primary acetogenic bacteria, which are all gram-negative bacteria, such as *Bacteroidales BS11 gut group unclassified, Succinivibrionaceae UCG-002, Prevotella, Prevotellaceae UCG-001, Prevotellaceae UCG-003* and *Anaeroplasma*, etc., have a significant negative correlation with the rumen LPS concentration, propionate ratio, butyrate ratio, etc. Moreover, the primary propionate-producing bacteria, which are all gram-positive bacteria, such as *Erysipelotrichaceae unclassified, Ruminococcus 2, Candidatus Saccharimonas* etc., have a significant negative correlation with the rumen pH, acetate ratio etc. These microbial results are also consistent with the differences in rumen fermentation indicators.

According to the results of the beta diversity analysis, there are also significant differences in the rumen bacterial community between the two groups of donor dairy goats (Fig. 2C). Among the two groups of dominant bacteria, the SARA group showed significantly increased relative abundances of the Firmicutes and Actinobacteria, which have been shown to be the primary starch-degrading bacteria in the rumen, and they significantly decreased the abundance of the Bacteroidetes, which has been shown to be the primary cellulolytic bacteria in the rumen^30,31^. Based on these above observations, the present study has also real-time monitored the ruminal bacterial alteration, especially the dynamic changes of 29 common differential genera during the 6 h after feeding in the goats from the two groups. Notably, genera that negative with the ruminal pH but positive with the LPS concentration, such as *DNF00809*, *Candidatus Saccharimonas*, *Lachnospiraceae NK3A20 group*, *Erysipelotrichaceae unclassified*, *Corynebacterium 1*, *Ruminococcus 2*, *Clostridiales unclassified*, were all significantly increased in SARA group among these 6 h. Although no previous study has reported the roles of these bacteria in SARA, these bacteria may serve as the key bacteria that led to the decrease of ruminal pH and occurrence of ruminal SARA. Furthermore, according to the real-time monitoring of the ruminal bacteria, the genera of *Prevotella*, *Prevotellaceae UCG-001*, *Prevotellaceae UCG-003*, and *Prevotella 1*, which were previously reported to respond for starch digestion, were significantly decreased in SARA (high starch diets) group among these 6 h. This result was mainly induced by the lysis of these bacteria when ruminal pH was lower than 5.6 for more than 3 h, which could further lead to the increase of ruminal free LPS and then induce the SARA occurrence^8,9^. Together, it can be concluded that with the increase of starch supplementary feeding, the ruminal pH was significantly decreased and the ruminal LPS concentration was significantly increased of the SARA group, which might influenced by these above differential genera but need further tested in the future study. This evidence suggests that there is a large difference in the microbial composition and fermentation of rumen fluid between the two groups of donors, and both can be used as important indicators of the two physiological conditions. Therefore, we hypothesize that the rumen microbiota from different donors can transfer the corresponding phenotypic to the intestines of antibiotic-treated mice, which was tested using rumen microbiota transplantation.

After RMT, there were significantly increased body weight gains compared with the treatment group without RMT. In view of the important role of intestinal microbes in the body’s digestion and use of nutrients, and according to the results of the colonic microbial alpha diversity after RMT in mice, the diversity and richness of microbes have increased compared with those without RMT^24^, ultimately improving the use of nutrients in mice. Moreover, we also found that among the mice that consumed high starch diets after RMT, the mice receiving SARA group rumen fluid had more significant weight gain than those receiving healthy group rumen fluid. This finding may be due to the large amount of starch-degrading bacteria in the rumen fluid of the SARA group, which can use high starch diets. The healthy group contains a lot of fibre-degrading bacteria, but it did not appear that the mice who received healthy group rumen fluid had more significant weight gain than those receiving SARA group rumen fluid through the high-fibre diet. This finding may be due to the low pH of the mouse stomach, resulting in the lysis of a large number of fibre-degrading bacteria and the inability to use nutrients in high-fibre diets.

By sequencing the microbial composition of the small intestine and colon contents of mice after RMT, we found that the microbial composition of the mouse colon contents was more similar to the donor rumen microbial composition. Similar to the identified significant differences of ruminal acetate and total acid concentration and proportion between goats from Health and SARA groups, only the composition of the VFA in the colon content was significantly changed between mice from Anti-Health-S and Anti-SARA-S groups (Fig. 6C–F). A large amount of microorganisms existed in the colon to ferment the undigested nutrients such as carbohydrates and proteins^32,33^, and studies have shown that the carbohydrates that escaped from the anterior intestine enter the colon and fermentation can reduce the blood sugar response, which is beneficial to the health of the host^34^. In addition, after mouse RMT, the mRNA expression of colonic inflammatory factors in the Anti-SARA-S group was significantly increased, and after RMT, the mRNA expression of Claudin-7 in the mouse colon was decreased. Because high-fibre diets can alleviate ruminant acidosis in ruminants, we conducted similar experiments on mice. The RMT from SARA goat to mice recipient who received high starch feed could significantly increase inflammatory response and decrease intestinal barrier function of mice colon when compared with mice recipient who receives microbiota from Healthy goat and high starch feed. However, although the same RMT experiment was performed, high-fibre treatment alleviated the inflammatory response and reduced the intestinal barrier damage of the colon of mice that receive the RMT from dairy goats in the SARA group. The differential results of these two experiments indicated that those bacteria linked to the increased of inflammatory were mainly the starch using bacteria, so that the high-fibre diet was not conducive to the growth of these bacteria and therefore alleviated the inflammatory response and reduced the intestinal barrier damage of the colon in mice. Just like the donor, high-grain diets can increase these starch using bacteria’s abundance, decrease ruminal pH, and then reduce the barrier function of the epithelia. These alteration increases the translocation of these endotoxins and other immunogenic compounds out of the digestive tract and may be the cause of inflammations in ruminants. And it can be prevented by balancing the diet with physically effective fibre, non-fibre carbohydrates and starch^35^, which is mainly due to the increase of fibre using bacteria, the following increase of ruminal pH, the decrease of lysis of starch using bacteria, and the decrease of ruminal LPS concentration. Furthermore, the inflammation in the colon of mice may also be directly caused by the high concentration of LPS in the rumen fluid of the donors from the SARA group^36,37^, and the application of dietary fibre has been widely shown to reduce colon inflammation and epithelial permeability^38^.

There are some limitations of the present study; first, the germ-free mice and antibiotic-treated mice were both widely used to test the roles of intestinal microbiota, while only antibiotic-treated mice were used as RMT recipients in this experiment. There are still large differences between antibiotic-treated mice and the germ-free mouse model, so in similar future experiments, germ-free mice could be considered for selection as the recipients. In addition, the content of mouse samples is lower, which makes it impossible to measure some indicators corresponding to the donor, such as the pH value of the intestinal contents. Further, the present study mainly focused on the roles of bacterial community alteration in the occurrence of SARA using the 16 S rRNA gene sequencing, the roles of other compositions of ruminal microbiota such as fungus and archaea cannot be well identified. Lastly, taxonomic classification is not very reliable at the species level by amplifying part of the 16 S rRNA gene (V3-V4 regions)^39^ and the use of PICRUSt2 could not reflect the actual metagenome and microbial function changes. However, comparing the previous version of PICRUSt, using PICRUSt2 could provide a better prediction to search for potential metagenome functional roles in SARA occurrence^40^. Herein, further experiments focused on the metagenome alteration using the shotgun sequencing could be performed to illuminate the roles of bacteria, fungus, and archaea in SARA occurrence.

There were several novel scientific projects that could be further studied based on our results. Considering the similar bacterial community of goats rumen were identified in the colon of mice recipient, a developed RMT approach through colon perfusion or rectal inoculation^41^ could be further tested in the future study and may serve as a feasible and effective method to test the ruminal microbial roles in mice model. Furthermore, the previous study has also reported the goats with acidosis susceptible or acidosis resistance when high grain diet was supplied^42^, which has not been studied in the present study. The illumination of its potential mechanism could help to further understand the host-microbiota interaction during the generating process of SARA, which was also worth further studied in the future.

In conclusion, there were significant differences in the bacterial community and rumen fermentation pattern between the two groups of dairy goat donors. After RMT, a fermentation pattern similar to that of the rumen was found in the colon, and the colonic microbiota was also closer to that of the donor. These findings show that the mouse colon can reflect the change in the rumen microbial composition to a certain extent. Simultaneously, RMT also transferred the inflammatory response of SARA to the mouse colon, and the application of high-fibre diet, which is similar to the SARA treatment in dairy goats, alleviated the colon inflammation in mice. This study provides basic data towards understanding the pathogenesis of subacute rumen acidosis, and it may provide a new perspective on using germ-free or antibiotics pre-treated mice as a model animal to verify the function of ruminant gastrointestinal microorganisms.

## Materials and methods

### Ethics approval statement

This experiment was conducted at the animal Research and Technology Center of Northwest A&F University (Yangling, Shanxi, China), and it was performed in accordance with the recommended guidelines from the Administration of Affairs Concerning Experimental Animals (Ministry of Science and Technology, China, revised 2004). The protocol was approved by the Institutional Animal Care and Use Committee at Northwest A&F University.

### Animals and experimental design

Twelve multiparous ruminally cannulated dairy goats with an average weight of ~50 kg were used in this study. The goats were fed a standard diet containing 70% forage and 30% concentrate mix, or a high-grain diet containing 30% forage and 70% concentrate mix (Table 1) ad libitum for 25 d, with an 18-d diet adaptation period and a 7-d data and sample collection period (Supplementary Fig. 7a). The goats were housed individually in their tie stalls, and they had free access to water. A total of 2 kg TMR experimental diet was fed to each goat twice daily at 0800 h and 1700 h. During the data and sample collection period, the pH values of the ruminal fluids were measured every hour for 6 consecutive hours after feeding in the morning every day (details are shown in the sample collection part below) to make sure the pH was lower than 5.6 for more than 3 h and the SARA of the dairy goat model was induced successfully. According to the different diets, dairy goats were randomly assigned to a healthy group (Health, concentrate to forage ratio (C: F) = 3: 7) and a subacute rumen acidosis group (SARA, C: F = 7: 3). These dairy goats had no history of gastrointestinal diseases or records of antibiotic use within 3 months.

**Table 1:** Ingredients and nutrient composition of the healthy and SARA groups on a dry matter (DM) basis.

Item	Treatment	
	Health	SARA
Ingredient (%)		
Corn	15.00	35.00
Corn germ meal	4.10	9.58
Corn gluten feed	7.50	17.50
Cottonseed meal	1.89	4.39
Corn silage	27.74	28.20
Alfalfa hay	42.26	1.80
Calcium phosphate	0.11	0.26
Limestone	0.68	1.59
Salt	0.24	0.56
Vitamin-mineral mix^1^	0.18	0.42
Sodium Bicarbonate	0.30	0.70
Nutrient composition		
DM^2^ (%)	52.16	51.64
NE_L_ (MJ/kg)	6.58	7.58
ADF (%)	21.6	12.33
NDF (%)	31.3	21.35
CP (%)	16.4	10.56
Starch (%)	28.4	54.53

^1^Vitamin-mineral mix (per kilogram): 450 mg of nicotinic acid, 600 mg of Mn, 950 mg of Zn, 430 mg of Fe, 650 mg of Cu, 30 mg of Se, 45 mg of I, 20 mg of Co, 800 mg of vitamin E, 45,000 IU of vitamin D, and 120,000 IU of vitamin A.

^2^DM, dry matter; NE_L_, net energy of lactation; ADF, acid detergent fibre; NDF, neutral detergent fibre; and CP, crude protein.

### Sample collection of dairy goats

On the second day of the ruminal microbe transplantation experiment (also recorded as the 23rd day of goat feeding experiment, Supplementary Fig. 7a), approximately 100 mL rumen fluid was further collected every hour for 6 consecutive hours after morning feeding, and it was strained through 4 layers of sterile cheesecloth. The pH of the rumen fluid was measured immediately with a mobile pH meter (HI 9024 C; HANNA Instruments, Woonsocket, RI, USA). Then, another 50 mL of strained rumen fluid was collected and stored at −80 °C to analyze the dynamic changes of the rumen microorganisms and rumen fermentation.

### Rumen fluid inoculum preparation

When the SARA of the dairy goat model was induced successfully, collected the rumen fluid 2 h after morning feeding^42^ for 3 days for inoculum preparation (Supplementary Fig. 7a). The rumen fluid inoculum was performed in accordance with Hu’s reports with some slight adjustments^43^. In brief, fresh rumen fluid was collected 2 h after morning feeding from dairy goat donors through the rumen fistula, mixed the rumen fluid of each group of dairy goats, and placed inside a sterile and anaerobic collection tube, and then transferred to the laboratory within one hour. The fluid was strained through four layers of sterile cheesecloth, then centrifuged at 6000 × *g* for 15 min. The precipitate without the supernatant was re-suspended in 1× PBS, and then, the resulting suspensions were transferred to the recipient mice directly. All the rumen fluid inoculum preparations were performed in an anaerobic incubator at a temperature of 37 °C.

### Murine intervention study

A total of 63 male Kunming (KM) mice weighing 18–20 g were obtained from the Laboratory Animal Center of the Fourth Military Medical University, and they were housed in cages in a specific pathogen-free animal facility at the College of Animal Science and Technology in Northwest A&F University. All the mice had *ad libitum* access to water and were kept under a 12/12 h light/dark cycle and at a 25 °C temperature during the entire experiment. After feeding with standard chow for a ten-day adaptation period, all the mice were randomly divided into 5 treatment groups, namely an S group (*n* = 6), Healthy-S group (*n* = 3), Anti-S group (*n* = 6), Anti-Health-S group (*n* = 24), and Anti-SARA-S group (*n* = 24). Briefly, the ‘S’ in the name of each group indicated the mice were fed with a high-starch (5% cellulose) diet, the ‘Anti’ in the name of each group indicated the mice were treated with antibiotics, and the ‘Health’ or ‘SARA’ means that the mice were inoculated with the ruminal microbiota of the corresponding dairy goat donor from ‘Health’ and ‘SARA’ groups, and a schematic overview of the experimental design is presented in Supplementary Fig. 7b. All the mice had *ad libitum* access to sterile standard chow, which comprised 83.7% carbohydrates, 12.9% protein, and 2.5% fat. First, the mice in the Anti-S, Anti-Health-S, and Anti-SARA-S groups were treated with ampicillin (1 g/L), ciprofloxacin (200 mg/L), and metronidazole (1 g/L), which were dissolved in the drinking water for 3 weeks^24^. Then the mice in the Anti-S, Anti-Health-S, and Anti-SARA-S groups were infused by intragastric gavage with 0.5 mL of high-concentration antibiotics once a day for 3 days, and the S group was supplied with sterile water. After a 24-h antibiotic-free period, the mice in the Anti-Health-S and Anti-SARA-S groups were infused by intragastric gavage with 0.3 mL of mixed rumen fluid derived from healthy dairy goats or SARA dairy goats for 3 days (Supplementary Fig. 7a, b) through the mouth by using 65 mm straight gavage needle, the other groups were given equal amounts of 1× PBS and maintained on a high-starch diet with ad libitum access to food for 10 days and then all the mice were sacrificed for the experiment.

Another 60 male Kunming (KM) mice weighing 18–20 g were obtained from the Laboratory Animal Center at the Fourth Military Medical University to investigate the effect of a high-fibre diet on inflammation in the mouse colon, and they were fed with high-fibre diets (50% cellulose) after RMT (Supplementary Fig. 7c). All the mice were randomly divided into 4 treatment groups, namely an F group (*n* = 6), Anti-F group (*n* = 6), Anti-Health-F group (*n* = 24), and Anti-SARA-F group (*n* = 24). Here, the ‘F’ in the name of each group indicated the mice were fed with a high-fibre diet. The other test procedures were the same as those given above.

### Sample collection of mouse recipients

All the mice were sacrificed on day 38, and they were weighed and then euthanized by exsanguination after the intravenous administration of 10% chloral hydrate solution (100 mg chloral hydrate/kg body weight; Sigma, USA) and immediately dissected. Blood samples were collected by eyeball enucleation, and the plasma samples were prepared and stored at −80 °C until the analyses. The liver, thymus, kidneys, and spleen were collected and weighed immediately. The organ indexes were expressed relative to the body weight (kg of organ/kg of body weight × 100). Each intestine segment was divided into two parts, one part was firstly used to collect intestinal contents instantly, and the other part was then used to collect intestinal tissue. Here, the contents of the duodenum, jejunum, and ileum were immediately collected and mixed as the small intestine contents, and the contents of the cecum and colon were immediately collected and stored directly. All contents were stored in liquid nitrogen for 24 h, and then transfer to -80 °C until further DNA extraction and intestinal fermentation detection. Then, the tissues (about 1 cm in length) from the duodenum, jejunum, ileum, and colon tissue whose contents were removed and washed with PBS buffer, were immediately stored in liquid nitrogen for 24 h, and then transfer to -80 °C until further RNA extraction.

### Chemical analysis of feedstuff samples

Samples of feed were dried at 55 °C for 72 h and then ground through a 1-mm screen. They were analyzed for their DM, ash, and crude protein contents according to the AOAC^44^, NDF, and ADF in Van Soest et al.^45^, with sodium sulfite and heat-stable α-amylase (Ankom® A200I fiber analyzer, ANKOM Technology, Macedon, NY, USA).

### Determination of volatile fatty acids (VFAs) in rumen fluid and intestinal contents

For the VFA measurements, the rumen fluid and mouse intestinal contents (dissolve each 0.5 g in 1 mL sterile water until completely dissolved) were centrifuged at 13,000 × *g* for 10 min, and then detected as previously described^8^. In brief, each 4 mL supernatant was mixed with 1 mL metaphosphoric acid (250 g/L), then centrifuged for 15 min at 10,000 × *g* at 4 °C. Two milliliters of the supernatant was mixed with 200 µL of crotonic acid (10 g/L) and then filtered through a 0.45 µm filter. The VFAs were separated and quantified with an Agilent 7820 A GC system equipped with a polar capillary column (AE-FFAP, 30 m × 0.25 mm × 0.33 μm) and a flame ionization detector (FID).

### Determining the LPS concentration of rumen fluid

Detailed procedures for the sample preparation and LPS determination have been described previously^46^. In brief, the rumen fluid samples, which were further heated at 100 °C for 30 min before being stored at −20 °C, were used for the LPS determination. The Limulus amoebocyte lysate (LAL) assay was used for LPS determination. The assay was performed using a 96-well microplate kit with an absorbance reading at 405 nm on a microplate reader (model 3550; Bio-Rad, Hercules, CA).

### Colonic epithelial RNA extraction and quantitative real-time PCR

The total RNA from colonic epithelial samples from all the mice was extracted using TRIzol reagent (Takara, Beijing, China). Specifically, DNaseI was used during the RNA isolation process to avoid contamination with genomic DNA. The quantity and purity of the total RNA were analyzed with a NanoDrop® ND-1000 spectrophotometer (Thermo Scientific, MA, USA), and the integrity of RNA was assessed by gel electrophoresis. Only RNA samples that had an OD260/280 > 1.8, OD260/230 > 2.0, and good integrity were used for further qRT-PCR.

Approximately 1 μg of total RNA from the intestinal epithelium was reverse-transcribed using the PrimeScript^TM^ RT reagent Kit with gDNA eraser (TaKaRa, Dalian, China). qRT-PCR was performed using SYBR® Green PCR Master Mix (TaKaRa, Dalian, China). A 20 μL PCR mixture was quickly prepared. Primers for *β-actin* (internal control genes) and the test mRNAs were designed using Primer-BLAST (http://www.ncbi.nlm.nih.gov/tools/primer-blast/) and are listed in Supplementary Table 2. In brief, the tested mRNAs included genes that were involved in inflammatory cytokine, i.e., *IL-1β*, *IL-6*, *TNF-α*, *IFN-γ*, *TLR-3*, and *TLR-4*, and genes of tight junction protein, *occluding, claudin-1, claudin-4, claudin-7*, and *ZO-1*. The PCR was conducted in an iCycler iQ5 multicolor real-time PCR detection system (Bio-Rad Laboratories) and programmed as follows: 95 °C for 10 min, 40 cycles of 95 °C for 10 s, 60 °C for 30 s, 72 °C for 30 s, and 72 °C for 5 min^47^. All the samples were examined in triplicate. All the data were analyzed using the 2^−ΔΔCt^ method^48^.

### DNA extractions, PCR amplification, and 16 S rDNA sequencing

DNA from the rumen fluid of the dairy goats and the small intestinal colonic contents of the mice was extracted using an E.Z.N.A. ®Stool DNA Kit (D4015, Omega, Inc., USA) according to the manufacturer’s instructions. Nuclease-free water was used for the blank. The total DNA was eluted in 50 μL of elution buffer and stored in a −80 °C freezer until further library preparation and 16 S rRNA gene sequencing.

The bacterial hypervariable regions V3 and V4 of the 16 S rRNA gene were PCR-amplified using bacterial forward and reverse primers 341 (5′-CCTACGGGNGGCWGCAG-3′) and 805 (5′-GACTACHVGGGTATCTAATCC-3′), respectively^49^. PCR amplification was performed in a total volume of 25 μL of reaction mixture containing 25 ng of template DNA, 12.5 μL of PCR Premix, 2.5 μL of each primer, and PCR-grade water to adjust the volume. The PCR conditions to amplify the prokaryotic 16 S fragments consisted of an initial denaturation at 98 °C for 30 seconds; 32 cycles of denaturation at 98 °C for 10 s, annealing at 54 °C for 30 s, and extension at 72 °C for 45 s; and then a final extension at 72 °C for 10 min. The PCR products were confirmed by 2% agarose gel electrophoresis. Throughout the DNA extraction process, ultrapure water was used instead of a sample solution as a negative control to exclude the possibility of false-positive PCR results. The PCR products were purified with AMPure XT beads (Beckman Coulter Genomics, Danvers, MA, USA) and quantified with Qubit (Invitrogen, USA). After the PCR amplicon library was prepared, the size and quantity of the amplicon library were assessed on an Agilent 2100 Bioanalyzer (Agilent, USA) and with the Library Quantification Kit for Illumina (Kapa Biosciences, Woburn, MA, USA), respectively. The libraries were sequenced on the NovaSeq PE250 platform.

### Illumina sequencing data analysis

Paired-end reads were assigned to samples based on their unique barcode and truncated by cutting off the barcode and primer sequence. Paired-end reads were merged using FLASH^50^. Quality filtering was performed on the raw reads under specific filtering conditions to obtain high-quality clean tags according to fqtrim (v0.94), and the chimeric sequences were filtered using Vsearch (v2.3.4)^51^. After dereplication using DADA2^52^, we obtained feature table and denoised feature sequences, which are called amplicon sequence variants (ASVs). Here, the detailed indices regarding the sequencing results and quality were listed in Supplementary Table 3.

The alpha and beta diversity were calculated by normalizing the same sequences randomly. According to the SILVA (release 132) classifier, the feature abundance was normalized using the relative abundance of each sample^53^. The alpha diversity indices of Chao1 and Shannon that were applied to analyze the complexity of species diversity and beta diversity of different groups were calculated by QIIME2^54^. BLAST was used for the sequence alignment, and the feature sequences were annotated with the SILVA database for each representative sequence to determine the different taxonomies at phylum and genus levels.

Phylogenetic investigation of communities by reconstruction of unobserved states 2 (PICRUSt2) analysis (https://github.com/picrust/picrust2)^40^ was used to predict the metagenome in the samples, and then the metagenome functions were predicted and the data were exported into levels 1 and 2 of the Kyoto Encyclopedia of Genes and Genomes (KEGG) database pathways.

### Statistical analysis

The statistical evaluation of ruminal fermentation and LPS contents of goats, as well as the intestinal fermentation parameters of mice, were analyzed by Students’ t-test using SPSS 21.0. After testing the normality and variance homogeneity of data, the statistical evaluation of growth performance, organ indices, and colonic epithelial gene expression of mice were analyzed by ANOVA test using SPSS 21.0. The statistical evaluation of ruminal pH alteration of the whole 6 h after morning feeding was analyzed by One-way Repeated Measures ANOVA procedure (the repeated measures analysis in the general linear model procedure) using SPSS 21.0. If a significant treatment effect was observed by ANOVA, the significant difference between treatments was identified by Duncan’s multiple comparisons test. All the data are expressed as the means with the standard error. Differences were considered to be statistically significant at *P* < 0.05.

The taxon abundance for each sample was determined according to the phylum, class, order, family, and genus. The Mann-Whitney U test was performed to compare the microbial alpha diversity between 2 compared groups. The Kruskal-Wallis test with Dunn’s post-hoc test was employed to test the microbial alpha diversity differences among 3 compared groups. The bacterial community was compared for their beta diversity using the distance matrices generated from the principal coordinated analysis (PCoA) and ANOSIM analysis based on the weighted UniFrac distance. The Mann-Whitney U test was used with multiple comparisons adjusted by the Benjamini–Hochberg false discovery rate (FDR) to rank bacteria that were significantly different in their genus/species levels and for predicted metagenome pathways analysis. Correlations between variables were tested by Pearson correlation test and meanwhile visualized by using corrplot and pheatmap R packages^55^.

### Reporting summary

Further information on research design is available in the Nature Research Reporting Summary linked to this article.

## Supplementary information

Supplementary Information

Reporting Summary

## Data Availability

All the data generated or analyzed in this study are included in this paper. The sequencing reads have been submitted and are available in the Sequence Read Archive (SRA) of NCBI under accession project number PRJNA662847.
