# Supplementary material for: Real-time monitoring of ruminal microbiota reveals their roles in dairy goats during subacute ruminal acidosis
Source: NPJ Biofilms Microbiomes. 2021 May 14;7:45. doi: 10.1038/s41522-021-00215-6 (PMC8121909; doi:10.1038/s41522-021-00215-6)
Supplement: Supplementary file 1 — Supplementary Information [file 41522_2021_215_MOESM1_ESM.pdf]

Real-time monitoring of ruminal microbiota reveals their roles in dairy goats during subacute ruminal acidosis

Xiaodong Chen[1], Xiaodong Su[1], Jilong Li[1], Yuntian Yang[1], Peiyue Wang[1], Fang Yan[1], Junhu Yao[1 *], and Shengru Wu [1, 2 *]

[1] College of Animal Science and Technology, Northwest A&F University, Yangling, Shaanxi, China

[2] Center for Translational Microbiome Research, Department of Molecular, Tumor and Cell Biology, Karolinska Institutet, Stockholm, Sweden

[*] **Co-corresponding authors:**

Junhu Yao, email: yaojunhu2004@sohu.com; Tel.: +86 13891883031；

Shengru Wu, email: wushengru2013@163.com; Tel.: +86 18700943648；

Supplementary Material

Supplementary Figure 1. The proportion of (A) valerate and (B) isovalerate from the rumen fluid of two groups of dairy goats 6 hours after feeding. All the data were expressed as the means with the standard error.

Supplementary Figure 2. (A) The differential bacteria at the phylum level (FDR < 0.05) and (B) the differential bacteria at the genus level (FDR < 0.05) between SARA and the healthy groups. The Mann-Whitney U test was used with multiple comparisons adjusted by the Benjamini–Hochberg FDR to identify significantly different bacteria. All bacteria listed here were all significantly changed bacteria with *FDR* < 0.05 between SARA and Healthy groups. All the data were expressed as the means with the standard deviation.

Supplementary Figure 3. The Chao 1 index and the Shannon index of rumen microbiomes from two groups of dairy goat donors at different time points after feeding. A Mann-Whitney U test was carried out for comparing the two groups.

H1 and S1: Rumen fluid of two groups of dairy goats 1 hour after feeding.

H2 and S2: Rumen fluid of two groups of dairy goats 2 hours after feeding.

H3 and S3: Rumen fluid of two groups of dairy goats 3 hours after feeding.

H4 and S4: Rumen fluid of two groups of dairy goats 4 hours after feeding.

H5 and S5: Rumen fluid of two groups of dairy goats 5 hours after feeding.

H6 and S6: Rumen fluid of two groups of dairy goats 6 hours after feeding.

Supplementary Figure 4. Principal Coordinate Analysis (PCoA) on rumen microbiomes from two groups of dairy goat donors at different time points after feeding. The data were statistically analyzed based on ANOSIM analysis.

H1 and S1: Rumen fluid of two groups of dairy goats 1 hour after feeding.

H2 and S2: Rumen fluid of two groups of dairy goats 2 hours after feeding.

H3 and S3: Rumen fluid of two groups of dairy goats 3 hours after feeding.

H4 and S4: Rumen fluid of two groups of dairy goats 4 hours after feeding.

H5 and S5: Rumen fluid of two groups of dairy goats 5 hours after feeding.

H6 and S6: Rumen fluid of two groups of dairy goats 6 hours after feeding.

Supplementary Figure 5. Effects of antibiotics treatments and RMT on the mouse intestinal microbial composition. (A) The Chao 1 index and (B) the Shannon index of the small intestine bacterial community of mice after antibiotics treatment. (C) The Chao 1 index and (D) the Shannon index of the colonic bacterial community of mice after antibiotics treatment. A Mann-Whitney U test was carried out for comparing the two groups in (A-D). (E) Principal Coordinates Analysis of the small intestinal bacterial community of mice after antibiotics treatment. (F) Principal Coordinates Analysis of the colonic bacterial community of mice after antibiotics treatment. The data of (E) and (F) were statistically analyzed based on ANOSIM analysis. (G) The changes in the number of ASVs in the small intestinal bacterial community of mice after antibiotics treatment. (H) The changes in the number of ASVs in the small intestinal bacterial community of mice after antibiotics treatment. (I) Principal Coordinates Analysis of the small intestinal bacterial community after RMT with or without antibiotics pre-treatment. (J) Principal Coordinates Analysis of the colonic bacterial community after RMT with or without antibiotics pre-treatment.. The data of (I) and (J) were statistically analyzed based on ANOSIM analysis. Here, the DNA samples of one mouse from the

Health_S group could not pass the quality control process before sequencing, so we only have 2 replicates for Health_S group.

Anti: mice taking antibiotics, S: High starch diet, Health: mice infused by intragastric gavage with rumen fluid from healthy dairy goats, s: small intestine of mice, and c: mice colon.

Supplementary Figure 6. The microbial weighted UniFrac ANOSIM distances between colon or small intestinal of mice recipients and rumen of goats donors (A-B), and the VFAs concentration and relative proportion alteration in ceca or small intestine of mice recipients. (A) The microbial weighted UniFrac ANOSIM distances between colon or small intestinal of mice recipients and rumen of goats donors from Health group. (B) The microbial weighted UniFrac ANOSIM distances between colon or small intestinal of mice recipients and rumen of goats donors from SARA group. The data of (A-B) were statistically analyzed using the Kruskal-Wallis test with Dunn's post-hoc test. The concentration (C) and relative (D) proportion of VFA in ceca from antibiotic-treated mice fed with a high starch diet after RMT. The concentration (E) and relative proportion (F) of VFA in small intestines from antibiotic-treated mice fed with a high starch diet after RMT. All the data were expressed as the means with the standard error.

Notes: For mice groups name, Anti: mice taking antibiotics, Health: mice infused by intragastric gavage with rumen fluid from healthy dairy goats, SARA: mice infused by intragastric gavage with rumen fluid from SARA dairy goats; S: mice fed with high starch diet after RMT, and F: mice fed with high fibre diet after RMT.

a-c the different superscript letters indicated a significant difference ($P < 0.05$).

Supplementary Figure 7. Schematic diagram of the experimental design of goat (A) and mice who received high starch diet (B) or high fibre diet (C). Note: For mice group names in panel (B and C), Anti: mouse taking antibiotics, Health: infused by intragastric gavage with rumen fluid from healthy dairy goats, SARA: infused by intragastric gavage with rumen fluid from SARA dairy goats; S: high starch diet, and F: High fibre diet.

Supplementary Table 1. Effects of the body weight, organ weight and organ index of mice fed a high starch diet after RMT

[a-b] Mean values within a row with the same superscript letters indicated no significant difference.

Supplementary Table 2. The primers of the genes tested in this study

Supplementary Table 3 The statistics of obtained sequences data of 16S RNA gene sequencing in the present study.

Supplementary Material:

Supplementary Figure 1:

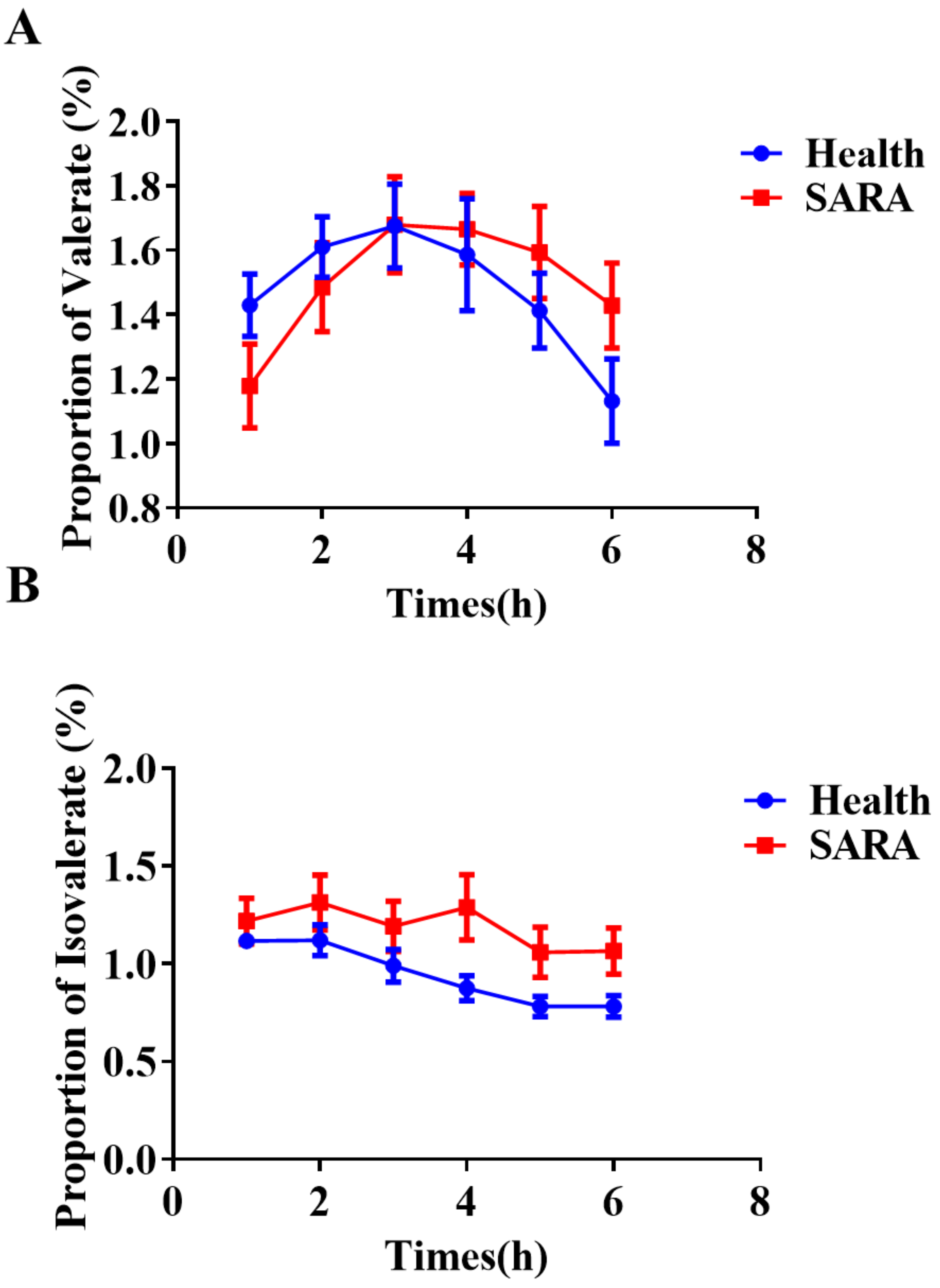

Supplementary Figure 2:

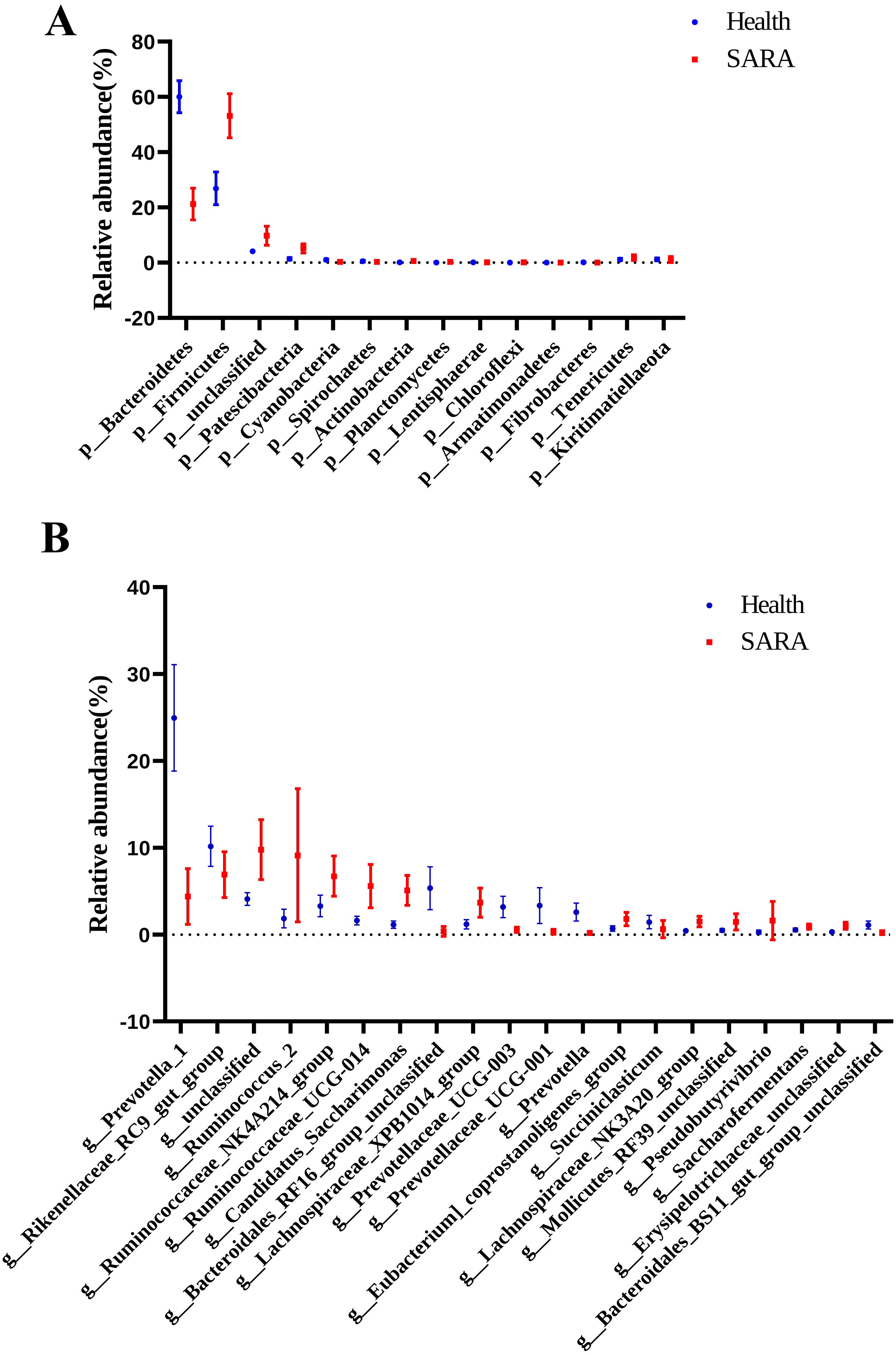

Supplementary Figure 3:

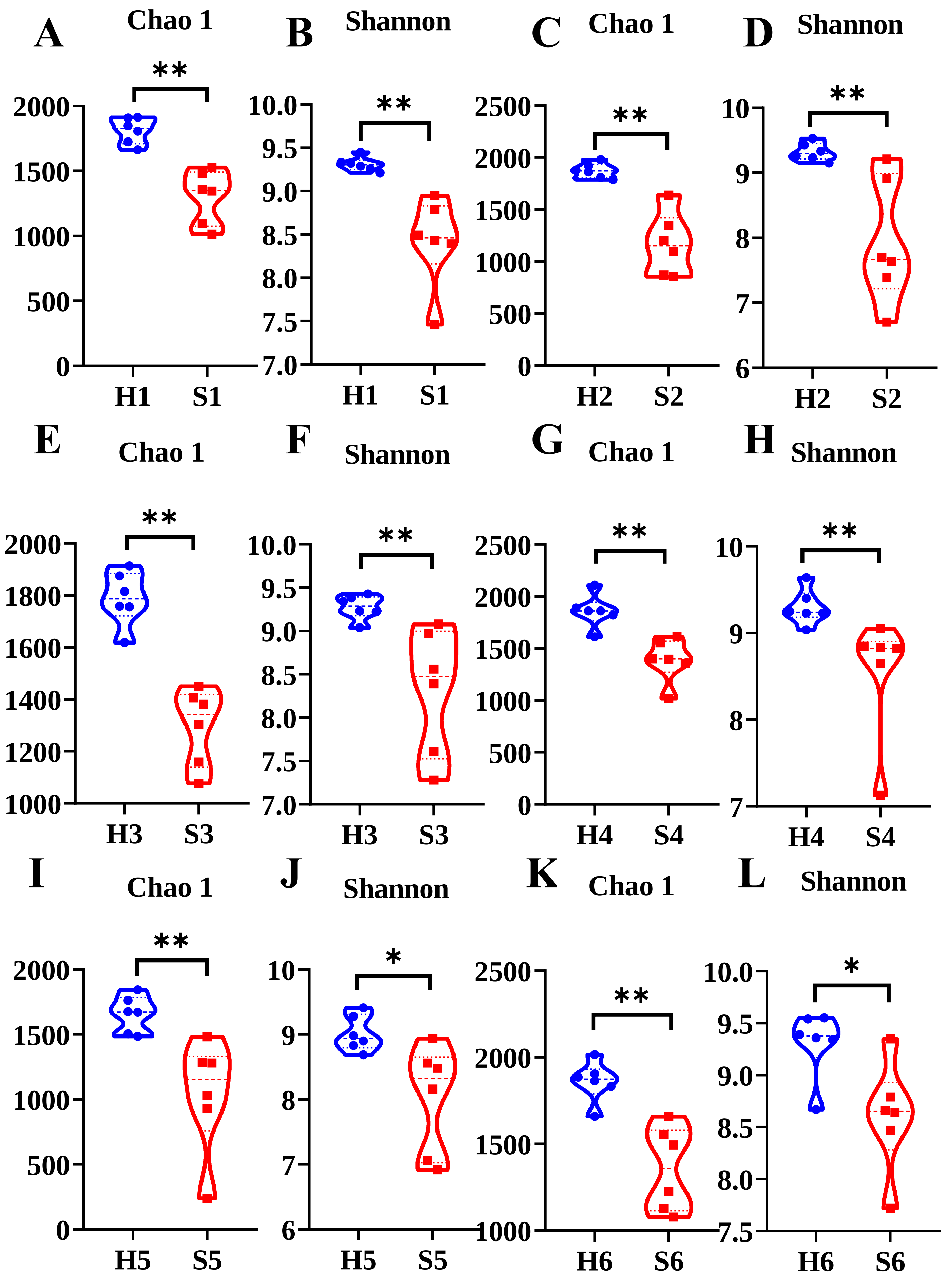

Supplementary Figure 4:

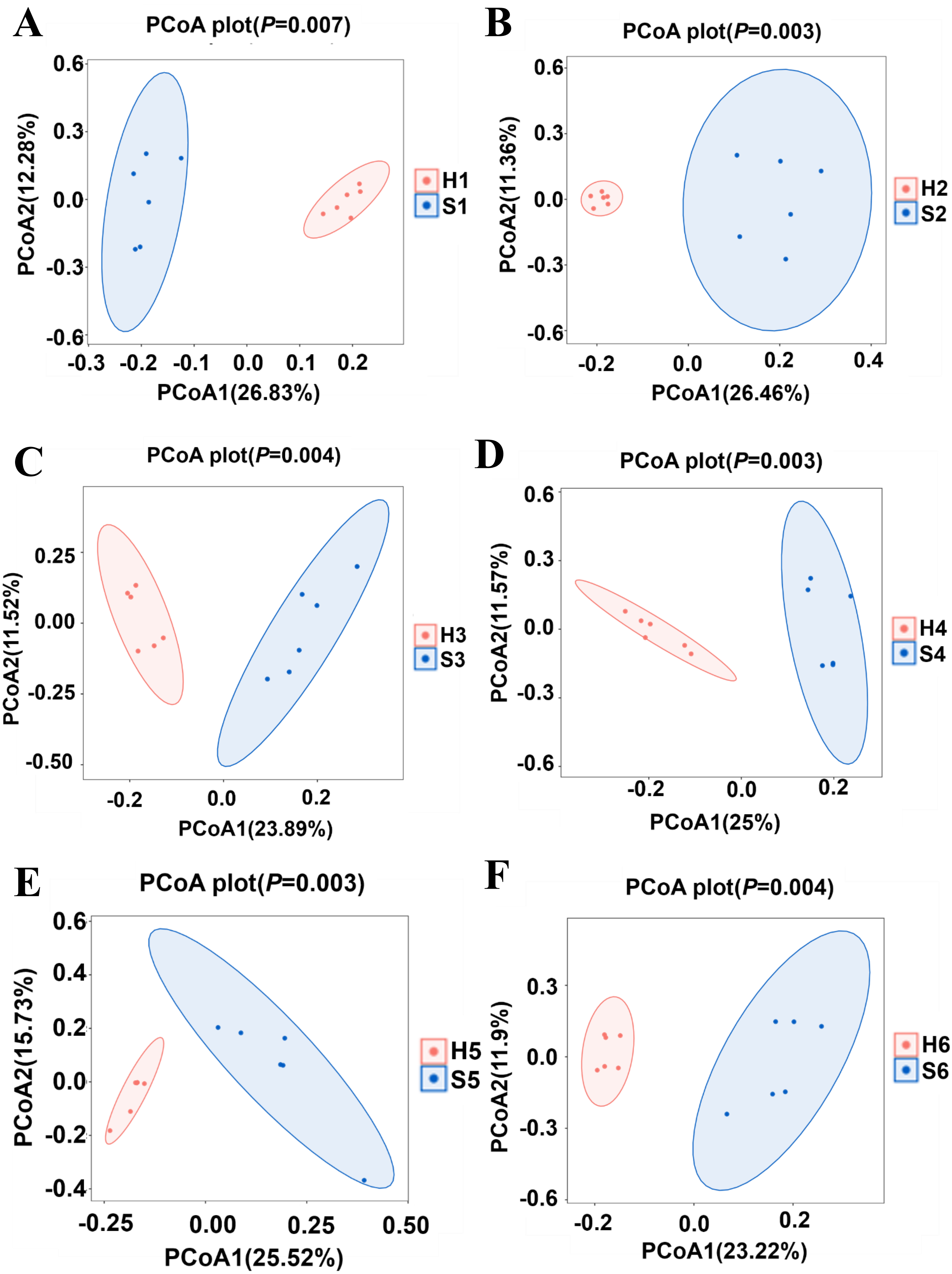

Supplementary Figure 5:

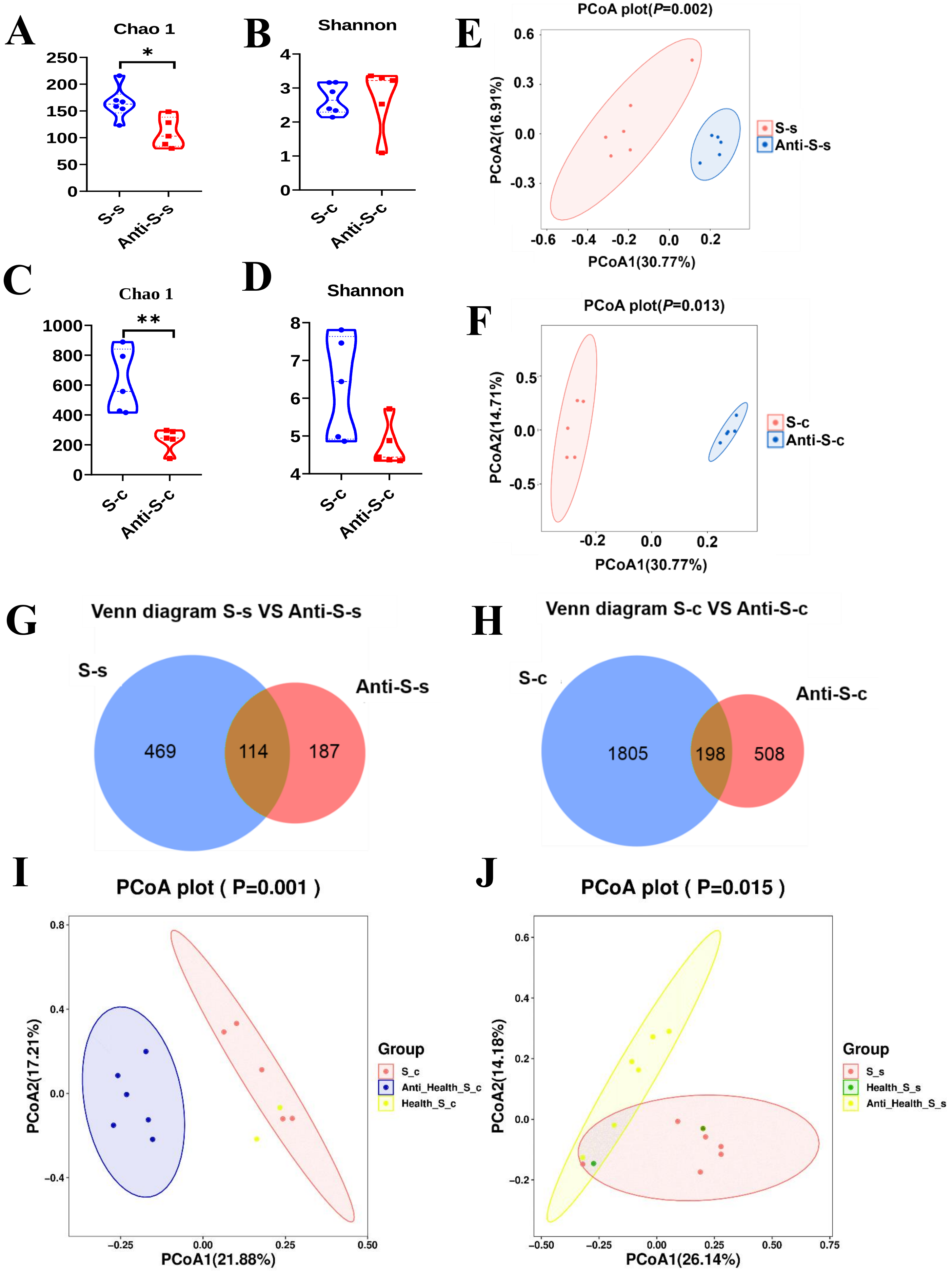

Supplementary Figure **6:**

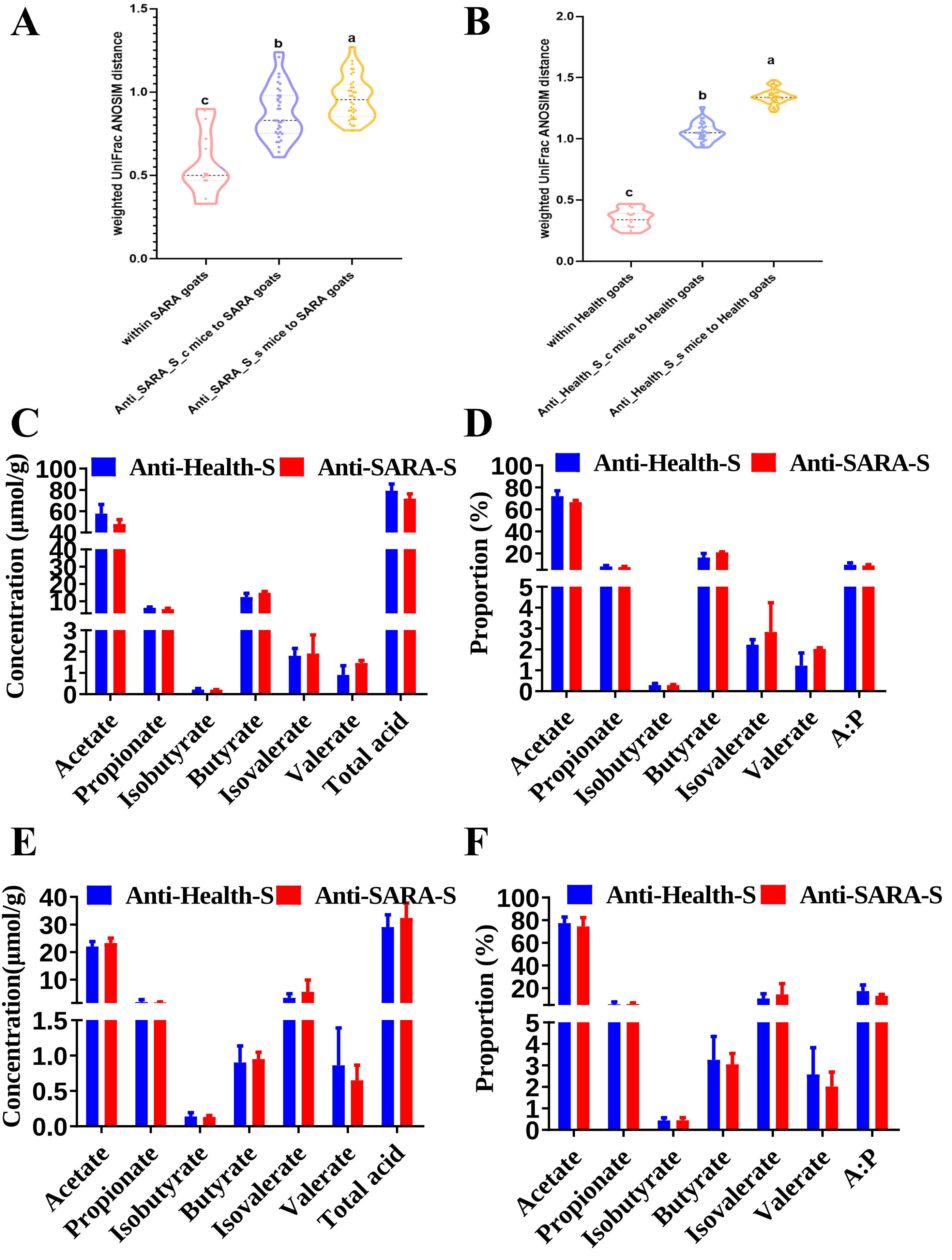

Supplementary Figure 7:

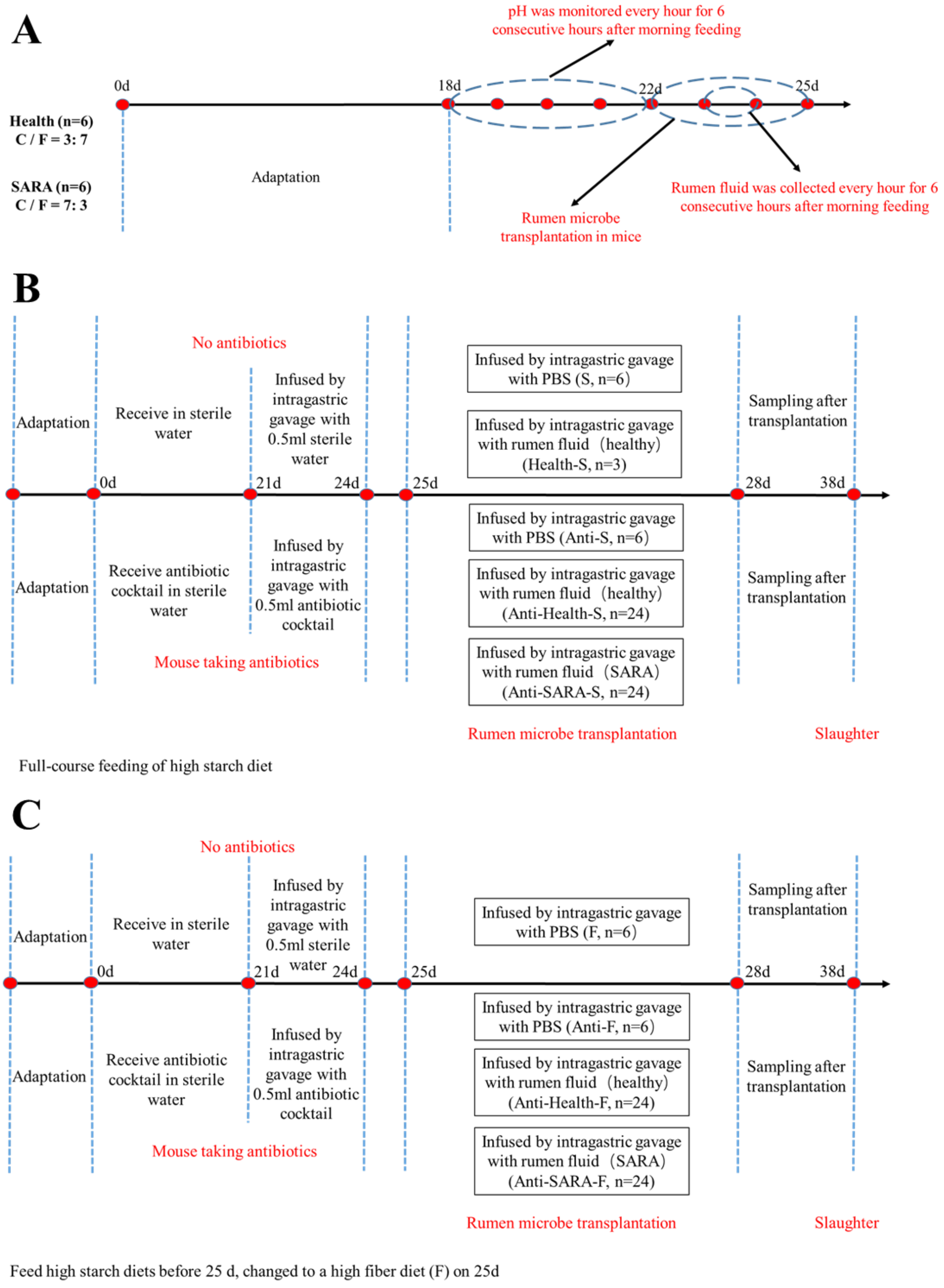

Supplementary Table 1 Effects of body weight, organ weight and organ index of mice fed high starch diet after RMT

Items	Treatments			SEM	*P*-value
	Anti-S	Anti-Health-S	Anti-SARA-S		
Body weight/g	44.85	45.85	45.69	0.538	0.837
Body weight gain/g	1.20[b]	1.25[b]	2.58[a]	0.260	0.032
Liver weight /g	2.27	2.02	2.10	0.044	0.137
Pancreas weight /g	0.19[b]	0.25[a]	0.21[ab]	0.009	0.015
Thymus weight /g	0.09	0.08	0.08	0.004	0.706
Spleen weight /g	0.10	0.12	0.11	0.004	0.152
Liver index	4.81	4.33	4.58	0.083	0.097
Pancreas index	0.41[b]	0.55[a]	0.43[b]	0.020	0.005
Thymus index	0.19	0.18	0.18	0.008	0.889
Spleen index	0.21	0.25	0.24	0.009	0.159

Supplementary Table 2 The primers of the genes tested in this study

Gene	PCR production	Primers sequences (5' to 3')	
IL-1β	220	F	TGCCACCTTTTGACAGTGATG
		R	AAGGTCCACGGGAAAGACAC
IL-6	76	F	TAGTCCTTCCTACCCCAATTTCC
		R	TTGGTCCTTAGCCACTCCTTC
TNF-α	148	F	CCTGTAGCCCACGTCGTAG
		R	GGGAGTAGACAAGGTACAACCC
IFN-γ	201	F	GCCACGGCACAGTCATTGA
		R	TGCTGATGGCCTGATTGTCTT
TLR-3	95	F	AAGTTATTCGCCCTCCTCTTGA
		R	AGATTCTGGATGCTTGTGTTTGA
TLR-4	222	F	CCTGACACCAGGAAGCTTGA
		R	TCAATTGTTTCAATTTCACACCTGG
occludin	117	F	CACACTTGCTTGGGACAGAG
		R	TAGCCATAGCCTCCATAGCC
claudin-1	137	F	TGGGGCTGATCGCAATCTTT
		R	CACTAATGTCGCCAGACCTGA
claudin-4	101	F	AAGTGCACCAACTGCATGGA
		R	ACGGGCACCATAATCAGCAT
claudin-7	238	F	GGCCTGATAGCGAGCACTG
		R	TGGCGACAAACATGGCTAAGA
ZO-1	*156*	F	GCTTTAGCGAACAGAAGGAGC
		R	TTCATTTTTCCGAGACTTCACCA
β-actin	*245*	F	GTGACGTTGACATCCGTAAAGA
		R	GCCGGACTCATCGTACTCC

Supplementary Table 3 The statistics of obtained sequences data of 16S RNA gene sequencing in the present study.

Group name	Sample name	Raw_Tags	Raw_Bases	Valid_Tags	Valid_Bases	Valid%	Q20%	Q30%	GC%
Mice 16S rRNA gene sequencing data									
S_s	S_s_1	87010	43.51M	70965	30.27M	81.56	97.48	92.81	50.87
	S_s_2	82114	41.06M	70081	29.84M	85.35	97.61	93.17	51.46
	S_s_3	81836	40.92M	67247	28.66M	82.17	96.53	90.70	50.63
	S_s_4	87869	43.93M	73447	31.31M	83.59	97.48	92.66	51.23
	S_s_5	85010	42.51M	69625	29.61M	81.90	97.25	92.24	51.36
	S_s_6	80700	40.35M	71925	30.47M	89.13	97.46	92.69	51.56
Health_S_s	Health_S_s_1	84584	42.29M	76806	32.68M	90.80	97.39	92.49	50.64
	Health_S_s_2	83541	41.77M	69015	29.41M	82.61	97.36	92.43	51.35
	Health_S_c_1	83927	41.96M	64932	27.59M	77.37	96.58	90.70	51.12
	Health_S_c_2	82287	41.14M	61762	25.85M	75.06	97.88	93.73	52.55
Anti_S_s	Anti_S_s_1	82407	41.20M	73360	30.89M	89.02	97.59	92.92	51.67
	Anti_S_s_2	84037	42.02M	73789	31.23M	87.81	97.15	92.02	52.12
	Anti_S_s_3	80567	40.28M	64394	27.14M	79.93	97.41	92.56	52.25
	Anti_S_s_4	82295	41.15M	69237	29.14M	84.13	93.76	84.33	53.57
	Anti_S_s_5	85614	42.81M	80292	34.21M	93.78	95.75	88.91	50.49
Anti_Health_S_s	Anti_Health_S_s_1	82415	41.21M	78533	33.43M	95.29	97.48	92.69	51.10
	Anti_Health_S_s_2	81356	40.68M	78095	33.25M	95.99	96.96	91.46	50.91
	Anti_Health_S_s_3	80727	40.36M	77807	33.12M	96.38	97.31	92.27	50.89
	Anti_Health_S_s_4	85189	42.59M	75105	31.89M	88.16	97.74	93.40	52.11
	Anti_Health_S_s_5	85538	42.77M	76205	31.71M	89.09	95.87	88.64	52.61
	Anti_Health_S_s_6	85217	42.61M	70657	29.82M	82.91	98.00	93.94	53.24
Anti_SARA_S	Anti_SARA_S_s_1	81700	40.85M	69870	29.82M	85.52	96.09	89.21	51.20

	Anti_SARA_S_s_2	85202	42.60M	73758	31.06M	86.57	97.82	93.50	52.10
	Anti_SARA_S_s_3	85306	42.65M	78250	32.50M	91.73	97.91	93.65	54.56
	Anti_SARA_S_s_4	84519	42.26M	79103	33.44M	93.59	97.40	92.53	51.73
	Anti_SARA_S_s_5	85455	42.73M	73106	30.57M	85.55	97.65	93.18	53.00
	Anti_SARA_S_s_6	86313	43.16M	77489	32.59M	89.78	97.16	91.91	51.91
S_c	S_c_1	84449	42.22M	66980	28.08M	79.31	97.64	93.14	51.94
	S_c_2	80346	40.17M	69723	29.05M	86.78	97.45	92.68	54.27
	S_c_3	86923	43.46M	73922	30.91M	85.04	96.23	89.92	52.56
	S_c_4	81212	40.61M	68741	28.80M	84.64	97.70	93.18	53.11
	S_c_5	86110	43.05M	72175	29.78M	83.82	97.60	93.04	54.12
Anti_S_c	Anti_S_c_1	83868	41.93M	65650	27.43M	78.28	96.32	89.99	51.48
	Anti_S_c_2	82609	41.30M	69659	29.47M	84.32	97.60	93.02	52.80
	Anti_S_c_3	80929	40.46M	66032	27.63M	81.59	97.13	91.91	54.68
	Anti_S_c_4	86225	43.11M	74267	31.06M	86.13	97.74	93.38	54.21
	Anti_S_c_5	80211	40.11M	68274	28.81M	85.12	97.44	92.73	53.71
Anti_Health_S_c	Anti_Health_S_c_1	80006	40.00M	67002	27.98M	83.75	94.96	86.91	52.70
	Anti_Health_S_c_2	87002	43.50M	77827	32.51M	89.45	97.06	91.51	54.54
	Anti_Health_S_c_3	80748	40.37M	69985	29.03M	86.67	97.62	93.03	54.47
	Anti_Health_S_c_4	85844	42.92M	71213	29.51M	82.96	97.88	93.65	54.33
	Anti_Health_S_c_5	80138	40.07M	70209	28.83M	87.61	97.84	93.56	54.04
	Anti_Health_S_c_6	84633	42.32M	75889	31.03M	89.67	92.36	81.73	53.68
Anti_SARA_S_c	Anti_SARA_S_c_1	82645	41.32M	66859	28.15M	80.90	97.55	92.90	53.45
	Anti_SARA_S_c_2	82911	41.46M	70487	28.88M	85.02	96.45	90.27	53.84
	Anti_SARA_S_c_3	85887	42.94M	72180	30.29M	84.04	97.89	93.72	54.16
	Anti_SARA_S_c_4	83720	41.86M	69159	28.58M	82.61	97.74	93.52	53.32
	Anti_SARA_S_c_5	83385	41.69M	68182	27.97M	81.77	97.75	93.46	53.64

	Anti_SARA_S_c_6	86689	43.34M	78437	31.93M	90.48	96.62	90.48	53.73
Goat 16S rRNA gene sequencing data									
H1	H1_1	80400	40.20M	70795	29.48M	88.05	97.46	92.68	53.17
	H1_2	85256	42.63M	72894	30.41M	85.50	97.47	92.65	53.17
	H1_3	84005	42.00M	73023	30.47M	86.93	97.39	92.42	53.12
	H1_4	84601	42.30M	73238	30.54M	86.57	97.44	92.66	53.10
	H1_5	87574	43.79M	77132	32.11M	88.08	97.57	92.99	53.22
	H1_6	86410	43.20M	76613	31.94M	88.66	97.61	93.11	53.38
H2	H2_1	86406	43.20M	74788	31.16M	86.55	97.75	93.30	53.36
	H2_2	83558	41.78M	72602	30.35M	86.89	97.84	93.54	53.15
	H2_3	83408	41.70M	72281	30.18M	86.66	97.50	92.78	52.79
	H2_4	83675	41.84M	73229	30.49M	87.52	97.89	93.74	53.02
	H2_5	86329	43.16M	76553	31.92M	88.68	97.74	93.35	53.15
	H2_6	81956	40.98M	72689	30.33M	88.69	97.92	93.79	53.39
H3	H3_1	83211	41.61M	70578	29.42M	84.82	97.42	92.38	53.31
	H3_2	81210	40.60M	70045	29.17M	86.25	97.68	93.23	53.32
	H3_3	83088	41.54M	73706	30.78M	88.71	97.27	92.21	52.75
	H3_4	87261	43.63M	77529	32.32M	88.85	97.45	92.76	53.10
	H3_5	87178	43.59M	77978	32.46M	89.45	97.54	92.88	53.12
	H3_6	81597	40.80M	72107	30.09M	88.37	97.62	93.11	53.37
H4	H4_1	80789	40.39M	69969	29.18M	86.61	97.82	93.54	52.92
	H4_2	81853	40.93M	71271	29.73M	87.07	97.99	93.94	53.29
	H4_3	80017	40.01M	71241	29.74M	89.03	97.63	93.17	52.96
	H4_4	87055	43.53M	77463	32.42M	88.98	97.81	93.56	53.03
	H4_5	85730	42.87M	76818	31.98M	89.60	97.76	93.41	52.86

	H4_6	82908	41.45M	74299	30.82M	89.62	97.94	93.84	53.21
H5	H5_1	85821	42.91M	75222	31.49M	87.65	97.38	92.42	52.42
	H5_2	85978	42.99M	74551	31.00M	86.71	97.56	92.91	53.22
	H5_3	82877	41.44M	74740	31.24M	90.18	97.51	92.86	52.69
	H5_4	87006	43.50M	77474	32.34M	89.04	97.51	92.87	53.21
	H5_5	84488	42.24M	76326	31.86M	90.34	97.50	92.78	52.86
	H5_6	82009	41.00M	73139	30.53M	89.18	97.63	93.17	53.19
H6	H6_1	86434	43.22M	75400	31.35M	87.23	97.67	93.18	52.78
	H6_2	80739	40.37M	71310	29.60M	88.32	97.87	93.66	53.30
	H6_3	85001	42.50M	76617	31.83M	90.14	97.46	92.71	52.86
	H6_4	87582	43.79M	79507	33.04M	90.78	97.75	93.41	53.00
	H6_5	84101	42.05M	75291	31.38M	89.52	97.45	92.74	52.84
	H6_6	82451	41.23M	73415	30.68M	89.04	97.95	93.94	53.39
S1	S1_1	84502	42.25M	68504	28.16M	81.07	95.18	87.36	53.22
	S1_2	85785	42.89M	74929	30.71M	87.35	97.61	92.81	52.61
	S1_3	87277	43.64M	73496	30.24M	84.21	95.87	89.08	53.15
	S1_4	84382	42.19M	74347	30.55M	88.11	97.86	93.61	52.72
	S1_5	80440	40.22M	71336	29.20M	88.68	97.40	92.54	52.84
	S1_6	82744	41.37M	73746	30.16M	89.13	98.01	93.97	53.09
S2	S2_1	81971	40.99M	63383	26.09M	77.32	95.59	88.25	52.75
	S2_2	83886	41.94M	68940	28.64M	82.18	96.73	90.58	52.65
	S2_3	84706	42.35M	72798	29.82M	85.94	97.75	93.39	53.02
	S2_4	85030	42.52M	74607	30.73M	87.74	96.38	90.01	52.80
	S2_5	83282	41.64M	73855	30.07M	88.68	97.97	93.96	52.57
	S2_6	84517	42.26M	76892	31.39M	90.98	97.36	92.32	53.08
S3	S3_1	82624	41.31M	68088	28.08M	82.41	96.99	91.60	53.05

	S3_2	87319	43.66M	72756	30.04M	83.32	98.09	94.12	52.59
	S3_3	86627	43.31M	77402	31.79M	89.35	96.78	91.21	53.34
	S3_4	82845	41.42M	74077	30.46M	89.42	97.80	93.48	52.48
	S3_5	85917	42.96M	77659	31.85M	90.39	96.75	90.97	52.86
	S3_6	80850	40.42M	74311	30.46M	91.91	97.08	91.58	53.10
S4	S4_1	86260	43.13M	72857	29.88M	84.46	97.65	93.09	52.75
	S4_2	87652	43.83M	73510	30.19M	83.87	97.21	92.02	52.73
	S4_3	85582	42.79M	75393	30.88M	88.09	97.39	92.48	52.91
	S4_4	84729	42.36M	75625	31.12M	89.26	96.85	91.25	52.54
	S4_5	86626	43.31M	78659	32.14M	90.80	97.98	93.97	52.49
	S4_6	81036	40.52M	74231	30.36M	91.60	97.14	91.82	52.89
S5	S5_1	80201	40.10M	64336	26.35M	80.22	90.67	78.97	53.27
	S5_2	80957	40.48M	70433	28.99M	87.00	97.70	93.15	52.62
	S5_3	84068	42.03M	73354	30.04M	87.26	96.31	90.04	52.81
	S5_4	83188	41.59M	73463	30.24M	88.31	97.74	93.33	52.35
	S5_5	87198	43.60M	77608	31.75M	89.00	94.46	86.18	52.38
	S5_6	86964	43.48M	79971	32.90M	91.96	97.99	93.95	53.19
S6	S6_1	83310	41.66M	71407	29.40M	85.71	97.64	93.06	53.04
	S6_2	80367	40.18M	71321	29.28M	88.74	96.49	90.20	52.50
	S6_3	80989	40.49M	73400	30.01M	90.63	97.78	93.51	52.48
	S6_4	85866	42.93M	77307	31.77M	90.03	96.67	90.73	52.29
	S6_5	84134	42.07M	75572	30.96M	89.82	97.77	93.49	52.12
	S6_6	82980	41.49M	74218	30.54M	89.44	96.94	91.29	53.32

Note: For mice data: the ‘S’ in the name of each group indicated the mice were fed with a high-starch (5% cellulose) diet, the ‘Anti’ in the name of each group indicated the mice were treated with antibiotics, and the ‘Health’ or ‘SARA’ means that the mice were inoculated with the ruminal

microbiota of the corresponding dairy goat donor from ‘Health’ and ‘SARA’ groups, the ‘s’ and ‘c’ indicated the content samples collected from small intestine and colon.

For goat data: H1, H2, H3, H4, H5, and H6: Rumen fluid of dairy goats from Health group that collected from 1, 2, 3, 4, 5, and 6 hours after morning feeding; S1, S2, S3, S4, S5, and S6: Rumen fluid of dairy goats from SARA group that collected from 1, 2, 3, 4, 5, and 6 hours after morning feeding.
