# Supplementary material for: Real-time monitoring of ruminal microbiota reveals their roles in dairy goats during subacute ruminal acidosis
Source: NPJ Biofilms Microbiomes. 2021 May 14;7:45. doi: 10.1038/s41522-021-00215-6 (PMC8121909; doi:10.1038/s41522-021-00215-6)
Supplement: Supplementary file 2 — Reporting Summary [file 41522_2021_215_MOESM2_ESM.pdf]

nature research

Corresponding author(s): Shengru Wu

Last updated by author(s): Jan 27, 2021

Reporting Summary

Nature Research wishes to improve the reproducibility of the work that we publish. This form provides structure for consistency and transparency in reporting. For further information on Nature Research policies, see our Editorial Policies and the Editorial Policy Checklist.

Statistics

For all statistical analyses, confirm that the following items are present in the figure legend, table legend, main text, or Methods section.

n/a	Confirmed	
[ ]	[x]	The exact sample size (*n*) for each experimental group/condition, given as a discrete number and unit of measurement
[ ]	[x]	A statement on whether measurements were taken from distinct samples or whether the same sample was measured repeatedly
[ ]	[x]	The statistical test(s) used AND whether they are one- or two-sided<br>*Only common tests should be described solely by name; describe more complex techniques in the Methods section.*
[ ]	[x]	A description of all covariates tested
[ ]	[x]	A description of any assumptions or corrections, such as tests of normality and adjustment for multiple comparisons
[ ]	[x]	A full description of the statistical parameters including central tendency (e.g. means) or other basic estimates (e.g. regression coefficient) AND variation (e.g. standard deviation) or associated estimates of uncertainty (e.g. confidence intervals)
[x]	[ ]	For null hypothesis testing, the test statistic (e.g. *F*, *t*, *r*) with confidence intervals, effect sizes, degrees of freedom and *P* value noted<br>*Give P values as exact values whenever suitable.*
[x]	[ ]	For Bayesian analysis, information on the choice of priors and Markov chain Monte Carlo settings
[x]	[ ]	For hierarchical and complex designs, identification of the appropriate level for tests and full reporting of outcomes
[ ]	[x]	Estimates of effect sizes (e.g. Cohen's *d*, Pearson's *r*), indicating how they were calculated

Our web collection on statistics for biologists contains articles on many of the points above.

Software and code

Policy information about availability of computer code

Data collection

1. The pH of the rumen fluid was measured immediately with a mobile pH meter (HI 9024C; HANNA Instruments, Woonsocket, RI, USA).
2.Samples of feed were dried at 55 °C for 72 h and then ground through a 1-mm screen. They were analyzed for their DM, ash, and crude protein contents according to the AOAC , NDF, and ADF in Van Soest et al. [44], with sodium sulfite and heat-stable α-amylase (Ankom® A200I fiber analyzer, ANKOM Technology, Macedon, NY, USA).
3. The VFAs were separated and quantified with an Agilent 7820A GC system equipped with a polar capillary column (AE-FFAP, 30 m × 0.25 mm × 0.33 µm) and a flame ionization detector (FID).
4. The Limulus amoebocyte lysate (LAL) assay was used for LPS determination. The assay was performed using a 96-well microplate kit with an absorbance reading at 405 nm on a microplate reader (model 3550; Bio-Rad, Hercules, CA).
5. the tested mRNAs included genes that were involved in inflammatory cytokine, i.e., IL-1β, IL-6, TNF-α, IFN-γ, TLR-3, and TLR-4, and genes of tight junction protein, occluding, claudin-1, claudin-4, claudin-7, and ZO-1. The PCR was conducted in an iCycler iQ5 multicolor real-time PCR detection system (Bio-Rad Laboratories) and programmed as follows: 95°C for 10 min, 40 cycles of 95°C for 10 s, 60°C for 30 s, 72°C for 30 s, and 72°C for 5 min.
6. 16S rDNA sequencing was performed on the NovaSeq PE250 platform.

Data analysis

Illumina sequencing data analysis
Paired-end reads were assigned to samples based on their unique barcode and truncated by cutting off the barcode and primer sequence. Paired-end reads were merged using FLASH. Quality filtering was performed on the raw reads under specific filtering conditions to obtain high-quality clean tags according to fqtrim (v0.94), and the chimeric sequences were filtered using Vsearch (v2.3.4). After dereplication using DADA2, we obtained feature table and denoised feature sequences, which are called amplicon sequence variants (ASVs).
The alpha and beta diversity were calculated by normalizing to the same sequences randomly. According to the SILVA (release 132) classifier, the feature abundance was normalized using the relative abundance of each sample. The alpha diversity indices of Chao1 and Shannon that were applied to analyze the complexity of species diversity and beta diversity of different groups were calculated by QIIME2. BLAST was used for the sequence alignment, and the feature sequences were annotated with the SILVA database for each representative sequence to

determine the different taxonomies at phylum and genus levels.
Phylogenetic investigation of communities by reconstruction of unobserved states 2 (PICRUSt2) analysis (https://github.com/picrust/picrust2) was used to predict the metagenome in the samples, and then the metagenome functions were predicted and the data were exported into levels 1 and 2 of the Kyoto Encyclopedia of Genes and Genomes (KEGG) database pathways.
Statistical Analysis
The statistical evaluation of ruminal fermentation and LPS contents of goats, as well as the intestinal fermentation parameters of mice, were analyzed by Students' t-test using SPSS 21.0. After testing the normality and variance homogeneity of data, the statistical evaluation of growth performance, organ indices, and colonic epithelial gene expression of mice were analyzed by ANOVA test using SPSS 21.0. The statistical evaluation of ruminal pH alteration of the whole 6 hours after morning feeding was analyzed by One-way Repeated Measures ANOVA procedure (the repeated measures analysis in the general linear model procedure) using SPSS 21.0. If a significant treatment effect was observed by ANOVA, the significant difference between treatments was identified by Duncan's multiple comparisons test. All the data are expressed as the means with the standard error. Differences were considered to be statistically significant at $P < 0.05$.
The taxon abundance for each sample was determined according to the phylum, class, order, family, and genus. The Mann-Whitney U test was performed to compare the microbial alpha diversity between 2 compared groups. The Kruskal-Wallis test with Dunn's post-hoc test was employed to test the microbial alpha diversity differences among 3 compared groups. The bacterial community was compared for their beta diversity using the distance matrices generated from the principal coordinated analysis (PCoA) and ANOSIM analysis based on the weighted UniFrac distance. The Mann-Whitney U test was used with multiple comparisons adjusted by the Benjamini–Hochberg false discovery rate (FDR) to rank bacteria that were significantly different in their genus/species levels and for predicted metagenome pathways analysis.
Correlations between variables were tested by Pearson correlation test and meanwhile visualized by using corrplot and pheatmap R packages.

For manuscripts utilizing custom algorithms or software that are central to the research but not yet described in published literature, software must be made available to editors and reviewers. We strongly encourage code deposition in a community repository (e.g. GitHub). See the Nature Research guidelines for submitting code & software for further information.

Data

Policy information about availability of data

All manuscripts must include a data availability statement. This statement should provide the following information, where applicable:

- Accession codes, unique identifiers, or web links for publicly available datasets
- A list of figures that have associated raw data
- A description of any restrictions on data availability

All the data generated or analyzed in this study are included in this paper. The sequencing reads have been submitted and are available in the Sequence Read Archive (SRA) of NCBI under accession project number PRJNA662847.

Field-specific reporting

Please select the one below that is the best fit for your research. If you are not sure, read the appropriate sections before making your selection.

☒ Life sciences ☐ Behavioural & social sciences ☐ Ecological, evolutionary & environmental sciences

For a reference copy of the document with all sections, see nature.com/documents/nr-reporting-summary-flat.pdf

Life sciences study design

All studies must disclose on these points even when the disclosure is negative.

Sample size	No statistical methods were used to predetermine sample size. All our data is from in vivo goat and mouse experiment: we always used as many mice and goats per group as possible in an attempt to minimize type I and type II errors. All sample sizes are indicated in the figures legends
Data exclusions	except fot the failed suquenced data, no data were excluded from the analysis.
Replication	The replications were described in the methods part. And on the graphs, individual dots represent individual samples used.
Randomization	All mice and goats with same sex, similar body weight were selected as the experimental animals, and then were randomly assigned to different groups.
Blinding	Investigators were blinded to allocation during experiments.

Reporting for specific materials, systems and methods

We require information from authors about some types of materials, experimental systems and methods used in many studies. Here, indicate whether each material, system or method listed is relevant to your study. If you are not sure if a list item applies to your research, read the appropriate section before selecting a response.

Materials & experimental systems

n/a	Involved in the study
☒	☐ Antibodies
☒	☐ Eukaryotic cell lines
☒	☐ Palaeontology and archaeology
☐	☒ Animals and other organisms
☒	☐ Human research participants
☒	☐ Clinical data
☒	☐ Dual use research of concern

Methods

n/a	Involved in the study
☒	☐ ChIP-seq
☒	☐ Flow cytometry
☒	☐ MRI-based neuroimaging

Animals and other organisms

Policy information about studies involving animals; ARRIVE guidelines recommended for reporting animal research

Laboratory animals

For Goats: Twelve multiparous ruminally cannulated dairy goats with an average weight of ~50 kg were used in this study. The goats were fed a standard diet containing 70% forage and 30% concentrate mix, or a high-grain diet containing 30% forage and 70% concentrate mix (Table 1) ad libitum for 25 d, with an 18-d diet adaptation period and a 7-d data and sample collection period (Figure S7A). The goats were housed individually in their tie stalls, and they had free access to water. A total of 2 kg TMR experimental diet was fed to each goat twice daily at 0800 h and 1700 h. During the data and sample collection period, the pH values of the ruminal fluids were measured every hour for 6 consecutive hours after feeding in the morning every day (details are shown in the sample collection part below) to make sure the pH was lower than 5.6 for more than 3 hours and the SARA of the dairy goat model was induced successfully. According to the different diets, dairy goats were randomly assigned to a healthy group (Health, concentrate to forage ratio(C：F) = 3: 7) and a subacute rumen acidosis group (SARA, C：F = 7: 3). These dairy goats had no history of gastrointestinal diseases or records of antibiotic use within 3 months.
For Mice: A total of 63 male Kunming (KM) mice weighing 18-20 g were obtained from the Laboratory Animal Center of the Fourth Military Medical University, and they were housed in cages in a specific pathogen-free animal facility at the College of Animal Science and Technology in Northwest A&F University. All the mice had ad libitum access to water and were kept under a 12/12 hours light/dark cycle and at a 25°C temperature during the entire experiment. After feeding with standard chow for a ten-day adaptation period, all the mice were randomly divided into 5 treatment groups, namely an S group (n=6), Healthy-S group (n=3), Anti-S group (n=6), Anti-Health-S group (n=24), and Anti-SARA-S group (n=24). Briefly, the 'S' in the name of each group indicated the mice were fed with a high-starch (5% cellulose) diet, the 'Anti' in the name of each group indicated the mice were treated with antibiotics, and the 'Health' or 'SARA' means that the mice were inoculated with the ruminal microbiota of the corresponding dairy goat donor from 'Health' and 'SARA' groups, and a schematic overview of the experimental design is presented in Figure S7B. All the mice had ad libitum access to sterile standard chow, which comprised 83.7% carbohydrates, 12.9% protein, and 2.5% fat. First, the mice in the Anti-S, Anti-Health-S, and Anti-SARA-S groups were treated with ampicillin (1 g/L), ciprofloxacin (200 mg/L), and metronidazole (1 g/L), which were dissolved in the drinking water for 3 weeks [24]. Then the mice in the Anti-S, Anti-Health-S, and Anti-SARA-S groups were infused by intragastric gavage with 0.5 mL of high-concentration antibiotics once a day for 3 days, and the S group was supplied with sterile water. After a 24-h antibiotic-free period, the mice in the Anti-Health-S and Anti-SARA-S groups were infused by intragastric gavage with 0.3 mL of mixed rumen fluid derived from healthy dairy goats or SARA dairy goats for 3 days (Figure S7A and S7B) through the mouth by using 65 mm straight gavage needle, the other groups were given equal amounts of 1× PBS and maintained on a high-starch diet with ad libitum access to food for 10 days and then all the mice were sacrificed for the experiment.
Another 60 male Kunming (KM) mice weighing 18-20 g were obtained from the Laboratory Animal Center at the Fourth Military Medical University to investigate the effect of a high-fibre diet on inflammation in the mouse colon, and they were fed with high-fibre diets (50% cellulose) after RMT (Figure S7C). All the mice were randomly divided into 4 treatment groups, namely an F group (n=6), Anti-F group (n=6), Anti-Health-F group (n=24), and Anti-SARA-F group (n=24). Here, the 'F' in the name of each group indicated the mice were fed with a high-fibre diet. The other test procedures were the same as those given above.

Wild animals

No wild animals were used in the study

Field-collected samples

No Field-collected samples

Ethics oversight

This experiment was conducted at the animal Research and Technology Center of Northwest A&F University (Yangling, Shanxi, China), and it was performed in accordance with the recommended guidelines from the Administration of Affairs Concerning Experimental Animals (Ministry of Science and Technology, China, revised 2004). The protocol was approved by the Institutional Animal Care and Use Committee at Northwest A&F University.

Note that full information on the approval of the study protocol must also be provided in the manuscript.
